# Colonization and population dynamics of total, viable, and culturable cells of two biological control strains applied to apricot, peach, and grapevine crops

**DOI:** 10.3389/fmicb.2023.1324965

**Published:** 2024-01-05

**Authors:** Núria Daranas, Esther Badosa, Emilio Montesinos, Anna Bonaterra

**Affiliations:** Institute of Food and Agricultural Technology-CIDSAV, University of Girona, Girona, Spain

**Keywords:** biological control agent, viability qPCR, *Bacillus velezensis*, *Lactiplantibacillus plantarum*, grapevine, peach, apricot

## Abstract

The ecological fitness of the biological control strains *Bacillus velezensis* A17 and *Lactiplantibacillus plantarum* PM411 was evaluated in different crops, geographical zones, and growing seasons. Both strains (2 g L^−1^ of dried formulation) were spray-inoculated on apricot trees, peach trees, and grapevines. Depending on the crop, flowers, fruits, and leaves were picked at several sampling time points. The population dynamics of viable, viable but non-culturable, and dead cells were studied by comparing viability qPCR (v-qPCR), qPCR, and plate counting estimations. A17 showed high survival rates in apricot, peach, and grapevine organs. The A17 viability was confirmed since qPCR and v-qPCR estimations did not significantly differ and were rather constant after field applications. However, higher population levels were estimated by plate counting due to the non-selective characteristics of the medium used. The viability of PM411 was constrained by plant organ, crop, and climate conditions, being higher in apricot than in grapevine. PM411 survival declined after field application, indicating difficulties in its establishment. The PM411 population level was made up of dead, culturable, and viable but non-culturable cells since significant differences between the three methods were observed. In conclusion, A17 and PM411 differ strongly in their survival in grapevine, peach, and apricot.

## 1 Introduction

Biological control of plant diseases is an effective and sustainable alternative or complement to chemical control. Although much research has been conducted to assess the potential of a wide range of microbial biological control agents (mBCAs), efforts are still required to increase the use and performance of commercially available microbial biopesticides. A wide variety of strains of bacterial genera, such as *Pseudomonas*, *Bacillus*, *Pantoea*, *Lactobacillus*, and *Streptomyces*, have been reported as mBCA of plant diseases against fungal and bacterial pathogens (Bonaterra et al., 2022).

*Bacillus velezensis* A17 and *Lactiplantibacillus plantarum* PM411 (formerly known as *Lactobacillus plantarum*) strains are two mBCA under development as plant protection products (PPPs). These two strains were selected among a large collection of strains of *Bacillus* spp. and lactic acid bacteria (LAB) in previous studies (Mora et al., 2011; Roselló et al., 2013).

*Bacillus velezensis* belong to the “operational group *B. amyloliquefaciens*” (Fan et al., 2017). *Bacillus* spp. are widely distributed in the soil, phyllosphere, and rhizosphere. In particular, the phyllosphere is a strongly competitive niche due to the challenging environment in relation to the discontinuous supply of nutrients, abiotic stresses (osmolarity, water availability, pH, radiation, etc.), and competition with cohabiting microorganisms (Harwood et al., 2018; Andric et al., 2020). The synthesis of a wide range of volatile organic compounds and bioactive secondary metabolites, including antimicrobial peptides and extracellular hydrolytic enzymes, contributes to the establishment and persistence of *Bacillus* spp. in the environment (Andric et al., 2020). For example, surfactins have a broad spectrum of physicochemical properties and biological activities (e.g., contributing to motility and biofilm formation), whereas the siderophore bacillibactin is involved in recovering trace elements from the environment (Andric et al., 2020). The hydrolytic enzymes, such as kinases, cellulases, or glucosidases, hydrolyze the major components of the fungal cell wall, whereas proteases, amylases, and cellulases help to recycle carbon and nitrogen (Harwood et al., 2018). In addition, the ability to produce endospores when facing harsh conditions allows *Bacillus* spp. to survive in various niches since they are highly resistant to environmental stresses, such as high temperature, irradiation, and exposure to solvents and enzymes (Ngalimat et al., 2021). *B. velezensis* A17 shows antagonistic activity against a wide range of both bacterial and fungal plant pathogens (Mora et al., 2015). In addition, A17 strain produces antimicrobial compounds, including cyclolipopeptides (bacillomycin, fengycin, iturin, and surfactin) (Mora et al., 2015), and triggers the defense response of plants, as reported in grapevine (Ramos et al., 2022).

*Lactiplantibacillus plantarum* is one of the most widespread species in its genus *Lactiplantibacillus* and is a facultative heterofermentative LAB that can be found in many different ecological niches, both in fermented foods and plant materials, as well as in the gastrointestinal tract of humans and animals (Siezen et al., 2010; da Silva Sabo et al., 2014). In fact, LAB are ubiquitous members of many plant microbiomes and are found in the phyllosphere, endosphere, and rhizosphere of many plants (Lamont et al., 2017). Each of these niches provides distinct challenges to the growth of the LAB (Lamont et al., 2017). The ability to inhabit different niches is associated with the capacity to ferment a wide range of carbohydrates (da Silva Sabo et al., 2014). Although LAB can survive under harsh environmental conditions, they have complex nutritional requirements and require several nutrients, including carbohydrates as energy and carbon sources, mineral salts, vitamins, amino acids, and nitrogenated bases for their growth (Wegkamp et al., 2010). They produce a variety of products including organic acids, amines, bacteriocins, vitamins, and exopolysaccharides during metabolism (Wang et al., 2021). *L. plantarum* PM411 is effective in preventing fire blight of apple and pear (*Erwinia amylovora*), bacterial canker of kiwifruit (*Pseudomonas syringae* pv. actinidiae), bacterial spot of stone fruits (*Xanthomonas arboricola* pv. pruni), and angular leaf spot of strawberry (*Xanthomonas fragariae*) (Roselló et al., 2017; Daranas et al., 2019). Its broad spectrum of antagonism is based on antimicrobial metabolites (organic acids, hydroxide peroxide, and proteinaceous compounds, such as bacteriocins), together with the colonization of plant surfaces (Roselló et al., 2013; Daranas et al., 2019).

The field performance of mBCA is considered valuable for the development of PPPs (Canfora et al., 2021). Therefore, the development of reliable and strain-specific monitoring methods is required (Manfredini et al., 2021). These methods must accurately detect and quantify the released mBCA in the environment to track its population dynamics over time, specifically in plant organs that are susceptible to being infected by pathogens to achieve satisfactory biocontrol of diseases. In addition, a specific monitoring method can also be appropriate to (i) optimize formulations and application strategies; (ii) assess environmental impact studies on native microbial communities; and (iii) evaluate post-release ecological fate and persistence, plant colonization, and residues (Gullino et al., 1995; Montesinos et al., 2002; Montesinos, 2003; Bonaterra et al., 2012; Manfredini et al., 2021).

Dilution-plate counting is the most used quantification method to estimate the population level of mBCA strains. This method is feasible when a selective growth medium is available (Usall et al., 2001; Nunes et al., 2002) or the target strain has unique and conspicuous phenotypic traits (i.e., antibiotic resistance or *gfp* reporter) (Bonaterra et al., 2005, 2007; Spinelli et al., 2005; Zhang et al., 2005; Stockwell et al., 2010). Nevertheless, in general, dilution-plate counting lacks specificity at the strain level since it does not permit the distinction between introduced strains and autochthonous wild populations of the same species or genus. Therefore, this may result in an overestimation of the mBCA population density (Badosa et al., 2004). In addition, considering that dilution-plate counting quantifies viable cells that are culturable, the viable population level may be underestimated using this approach due to the presence of viable but non-culturable (VBNC) cells (Oliver, 2005; Pinto et al., 2015).

Alternative non-culture-based methods have been implemented to study the dynamics of microorganism colonization in environmental samples. Most techniques are based on DNA amplification of the target; therefore, the extraction of community DNA is required. Then, the quantitative PCR (qPCR) is successfully applied due to its high strain specificity. For the development of a strain-specific qPCR method, it is necessary to find unique molecular markers for the corresponding strain. These strain-specific regions can be provided by next-generation sequencing (NGS) technologies (Hernández et al., 2020), genome mining and sequencing (Horn et al., 2016), whole-genome comparison between strains (Mendis et al., 2018), or even by DNA fingerprinting techniques, such as randomly amplified polymorphic DNA PCR (RADP), followed by sequence characterization becoming sequence-characterized amplified regions (SCAR) (Pujol et al., 2005; Felici et al., 2008; Von Felten et al., 2010; Gotor-Vila et al., 2016; Rotolo et al., 2016). qPCR approaches have been developed for specific monitoring of *Pseudomonas* spp., *Lactobacillus* spp., and *Bacillus* spp. strains on phyllosphere (Pujol et al., 2006; Daranas et al., 2018b), on fruit surfaces after harvesting (Soto-Muñoz et al., 2014b), and in soil environments (Felici et al., 2008; Von Felten et al., 2010; Mosimann et al., 2017). However, qPCR amplifies DNA from both viable and dead cells (Josephson et al., 1993). Therefore, a limitation of the conventional qPCR technique may be the overestimation (2–3 log CFU g^−1^) of viable cells as reported in previous studies on *L. plantarum* (Daranas et al., 2018b) and *Xylella fastidiosa* (Baró et al., 2020). In contrast, viability qPCR (v-qPCR) uses nucleic acid-binding dyes in combination with qPCR for selectively detecting and enumerating only viable cells (Codony et al., 2020). As reported in other bacteria, different nucleic acid-binding dyes, such as propidium monoazide (PMA or PMAxx^®^), ethidium monoazide (EMA), or PEMAX^®^, have been used in combination with qPCR (Randazzo et al., 2016; Sicard et al., 2019; Baró et al., 2020). The v-qPCR has been used for the detection and quantification of mBCA in greenhouse and post-harvest treatments (Soto-Muñoz et al., 2014a, 2015b; Daranas et al., 2018b), as well as in the field (Soto-Muñoz et al., 2015a). However, as far as we know, a strain-specific v-qPCR for the quantification of population levels of *Bacillus* or *Lactiplantibacillus* mBCA has not been developed and evaluated in apricot, peach, and grapevine under field conditions.

The aim of the present study was to evaluate and compare the ecological fitness of *B. velezensis* A17 and *L. plantarum* PM411 strains after their application to three plant crops (apricot, peach, and grapevine) and two geographic zones (Penedès zone in Spain and Pyrénées-Orientales department in France) over two growing seasons (2019, and 2020 or 2021). The field monitoring of both strains by strain-specific v-qPCR was compared with strain-specific qPCR and dilution-plate counting methods to differentiate the population level of dead, viable, and VBNC cells.

## 2 Materials and methods

### 2.1 Bacterial strains and growth conditions

The present study was focused on the biological control strains, such as *B. velezensis* A17 and *L. plantarum* PM411. The A17 strain was isolated from the flowers of *Lobularia maritima* (Mora et al., 2011), whereas the PM411 strain was isolated from the surface of a pear fruit (Trias et al., 2008). Both strains were deposited in the Colección Española de Cultivos Tipo (*L. plantarum* PM411, CECT 8299; *B. velezensis* A17, CECT 8836). A spontaneous mutant of wild-type *L. plantarum* PM411, resistant to rifampicin, obtained as previously described (Roselló et al., 2013), was used in this study. *B. velezensis* A17 was cultured on Luria–Bertani (LB) agar at 28°C for 24 h, and *L. plantarum* PM411 was grown on de Man–Rogosa–Sharpe (MRS) agar (Oxoid, Basingstoke, Hampshire, United Kingdom) at 28°C for 48 h. Both strains were stored in 20% glycerol at −80°C.

### 2.2 Production of *Bacillus velezensis* A17 and *Lactiplantibacillus plantarum* PM411

The fermentation processes were performed in a pilot-scale bioreactor (Biostat^®^ C, Sartorius AG, Göttingen, Germany) with a working volume of 30 L of production medium. For *B. velezensis* A17, the production medium (PM) consisted of modification of the original recipe of Walker and Abraham (1970) described by Ramos et al. (2022). For *L. plantarum* PM411, MRS broth was used, and the filter-sterilized solution of glucose was added after autoclaving the remaining components.

In total, 24 h old cultures of *B. velezensis* A17 in PM and *L. plantarum* PM411 in MRS broth obtained in two laboratory-scale bioreactors (New Brunswick BioFlo^®^/CelliGen^®^ 115, Eppendorf, Hamburg, Germany) were used as starter cultures to inoculate at a proportion of 10% of the 30 L bioreactor. The fermentation processes for *B. velezensis* A17 involved 48 h at 28°C with a pH of 7, whereas for *L. plantarum* PM411, it was 24 h at 30°C, with a pH of 6. The agitation ramp was set from 50 to 500 rpm to achieve 20% of dissolved oxygen. Antifoam (0.5 mL L^−1^) was added if needed.

After fermentation, the cells from cultures at the stationary phase were harvested. A continuous disc stack centrifuge (SA-1-02-175, GEA Westfalia, Granollers, Spain) was used at a working flow rate of 12 L h^−1^, rotation speed of 10,000 rpm, opening time of 1 s, and discharge time of 30 min. The concentrated cell suspension (10-fold), containing vegetative cells and spores, in the case of A17, together with secondary metabolites, was mixed with skimmed milk (A17) or lactose (PM411) at 15% final concentration, frozen at −70°C, and lyophilized in a laboratory-scale freeze-dryer (Unitop HL, VirTis, Gardiner, NY). Dried formulations were stored at 4°C in vacuum-sealed plastic-coated aluminum bags. For each lyophilized product, the cell concentration per gram (CFU g^−1^) was determined by plating 10-fold dilutions on LB or MRS agar.

### 2.3 Field trials

Over two growing seasons, 10 field trials were carried out in experimental orchards of apricot (“Royal”) and peach (“Corindon”) and in commercial organic vineyards (“Macabeu”) (Table 1).

**Table 1:** Location of orchard, experimental design, treatments, and doses of application in each field trial.

Trial code	Number of trials	Growing season	Crop	Variety	Location	Coordinates	Treatments^a^	Dose (Kg Ha^−1^)^b^	Volume (L Ha^−1^)
I/II	2	2019/2021	Apricot	“Royal”	Torreilles (France)	42.755179 N 2.981383 E	Non-treated control	–	300
							*L. plantarum* PM411	0.6	
							*B. velezensis* A17	0.6	
III/IV	2	2019/2020	Peach	“Corindon”	Torreilles (France)	42.7539503 N 2.97658681 E	Non-treated control	–	700
							*B. velezensis* A17	1.4	
V	1	2019	Grapevine	“Macabeu”	Sant Martí Sarroca (Spain)	41.384444 N 1.593611 E	Non-treated control	–	350
							*L. plantarum* PM411	0.7	
VI	1	2020			Vilafranca del Penedès (Spain)	41.333611 N 1.700833 E			
							*B. velezensis* A17	0.7	
VII/VIII	2	2019/2020			La Granada (Spain)	41.388333 N 1.745000 E	Non-treated control	–	200–400
							*L. plantarum* PM411	0.4–0.8	
							*B. velezensis* A17	0.4–0.8	
IX/X	2	2019/2020			Gelida (Spain)	41.450278 N 1.860833 E	Non-treated control	–	260–280
							*B. velezensis* A17	0.52–0.56	
							*B. velezensis* A17 + Copper oxychloride	0.52–0.56 + 0.8	

a Product concentration: 5×10^10^CFU g^−1^.

b Dose of application according to the phenological development stage and vegetation density of the crop.

Plots were distributed in a completely randomized block design with three (trials II, III, IV, V, VI, VIII, IX, and X) or four (trials I and VII) biological replicates per treatment. Each replicate consisted of three apricot or peach trees or ten grapevines. Buffer (non-treated) trees or vines were used to separate treatments and replicates. A weather station, placed in or near the experimental orchards (S.I.C.A. Centrex for trials I, II, III, IV; Sant Martí Sarroca for trial V, La Granada for trials VI, VII, VIII; Sant Sadurní d’Anoia for trials IX and X), recorded daily weather observations of temperature (°C), relative humidity (RH; %), and rainfall (mm).

The treatments performed consisted of *B. velezensis* A17 and *L. plantarum* PM411 applied individually. Both treatments were prepared at 2 g L^−1^ of dried formulation (5×10^10^ CFU g^−1^) in water to obtain a final concentration of 1×10^8^ CFU ml^−1^ (Table 1). A non-treated control was included in each trial. Treatment applications were conducted according to the schedules shown in Supplementary Table S1 (Meier, 2018), considering the phenological development stage and vegetation density of each crop, in particular. Trees and vines were sprayed in the morning until runoff using an atomizer (SR430 STIHL, Madrid, Spain) with different treatments. Treatment applications of each trial were performed within the standard pest management adopted in the orchards. For each crop, two independent field trials were performed in two different growing seasons (from 2019 to 2021).

### 2.4 Microvinification of *Bacillus velezensis* A17- and *Lactiplantibacillus plantarum* PM411-treated grapes

Grape bunches from each treatment and replicate of trials V and VI presenting similar ripeness were visually selected, harvested, and transported to the winery for conducting microvinification in three replicate batches. First, grapes were crushed and pressed to obtain grapevine must. Then, sulfite was added (40 mg L^−1^), and the crushed juice was allowed to settle overnight. After racking off the lees, the clear juice was inoculated with selected wine yeast Jazz™ at 25 g hL^−1^ (Chr. Hansen Holding A/S, Hoersholm, Denmark). Fermentation was monitored by measuring density and quantity of residual sugar. Once the sugar was fermented, the wine was racked off the lees, cold-stabilized, and then filtered.

### 2.5 Population dynamics of *Bacillus velezensis* A17 and *Lactiplantibacillus plantarum* PM411

The population levels of A17 and PM411 strains were tracked after their release into grapevine, apricot, and peach crops under field conditions as well as in grapevine must and wine. In addition, population dynamics of A17 were also monitored on peach fruits during the post-harvest period, which were kept at 20°C.

Sample collection was performed at several sampling time points (Supplementary Table S1). Samples of 12 apricot flowers or immature fruits, 6 peach fruits, 2 grapevine berries, and 5 grapevine leaves were picked from each replicate and were treated for each trial. Plant material washing was obtained by homogenizing plant organs (1–5 g of apricot flowers or immature fruits, 10 g of leaves, and 5 g of berries or fruit peal) with 50 mM sterile PBS (pH 7.0) and 0.1% peptone (at the proportion of 1g/10 mL) in an orbital shaker (KS501 digital; IKA Labortechnik, Staufen, Germany) at 130 rpm for 30 min on ice bath.

The population levels of A17 and PM411 on flowers, fruits, and/or leaves were determined by three different approaches: strain-specific TaqMan-based qPCR, v-qPCR and dilution-plate counting. Grapevine must and wine were only analyzed by qPCR.

For *B. velezensis* A17, the strain-specific qPCR and v-qPCR assays were specifically developed in the present study. In the case of *L. plantarum* PM411, both methods were previously reported by Daranas et al. (2018b), and v-qPCR was updated in the present study using PMAxx^®^ reagent (Supplementary material).

#### 2.5.1 Strain-specific TaqMan-based qPCR and v-qPCR assays

Bacterial DNA extraction was carried out by different procedures according to the sample type. DNA from plant material washing was isolated according to the method described by Schmidt et al. (2008) with some modifications (Daranas et al., 2018b). From grapevine must and wine, DNA was extracted by the mechanical, enzymatic, and chemical procedures as described by Daranas et al. (2018b) and purified using the GeneJET Genomic DNA Purification kit, following the specific protocol for gram-positive bacteria (Thermo Fisher Scientific, Waltham, United States).

For the v-qPCR analysis, before DNA isolation, samples of plant material washing were treated with Propidium monoazide PMAxx^®^ (Biotium, Fremont, California, United States) at 20 μM, according to the optimized procedure described in Supplementary material. In brief, 10 μL of PMAxx^®^ stock solution (2000 μM) was added to 1 mL of sample, thoroughly mixed and incubated for 10 min in the dark at room temperature in an orbital shaker KS501 digital (IKA Labortechnik Staufen, Germany) at 130 rpm. Immediately, samples were photo-activated for 15 min with the PhAST Blue photoactivation system (GenIUL, Barcelona, Spain) set to 100% and transferred to DNA low-binding 1.5 mL tubes (Sarstedt, Nümbrecht, Germany). Finally, PMAxx-treated cells were collected by centrifugation at 13200×g for 10 min and washed with 50 mM sterile PBS (pH 7.0) under the same centrifugation conditions.

Primers and TaqMan probes used for both v-qPCR and qPCR analysis are presented in Supplementary Table S2. Probes were labeled with the 6-carboxyfluoresceine (FAM) reporter dye at the 5′ end and the 6-carboxytetramethylrhodamine (TAMRA) quencher dye at the 3′ end. Two non-template controls (NTCs), using water instead of genomic DNA and DNA isolated from plant material washing without A17 or PM411, and positive control with A17 or PM411 DNA were included in all qPCR runs. All reactions were performed in triplicate and were carried out in a QuantStudio 5 Real-time PCR system (Applied Biosystems, Foster City, United States). Amplification mixture and qPCR conditions for each TaqMan-based qPCR assay are presented in Supplementary Table S3.

The quantification of the population level of A17 and PM411 strains was performed by means of a standard curve of the corresponding plant material washing (apricot flowers, peach fruits, grapevine flowers, berries, and leaves), grapevine must, and wine specifically developed for each strain. Cell suspensions of A17 and PM411 were prepared in sterile distilled water at high concentration (10^8^ CFU ml^−1^) and diluted to appropriate concentrations with plant material washing, must, or wine. The cell concentration was checked by OD_600_ measure, considering that 0.6 and 0.3 correspond to 10^8^ CFU ml^−1^ for *Bacillus* and *Lactiplantibacillus*, respectively, and was confirmed by colony counts. The tested concentrations covered a 5-log range, from 1 × 10^2^ to 1 × 10^7^ CFU mL^−1^, and were treated or not with PMAxx, as described above, for v-qPCR and qPCR quantification, respectively. Ct values were plotted against the logarithm of the initial number of CFU ml^−1^, and standard curves were generated by a linear regression of the plotted points. Slopes were used to determine the amplification efficiency of each design from the equation E (%) = (10^−1/slope^ − 1) × 100. The equations of regression curves for the A17 and PM411 designs, as well as the amplification efficiencies and sensitivity, are presented in Supplementary Table S4 (Supplementary Figure S1).

The number of total and viable cells estimated by qPCR and v-qPCR, respectively, was obtained by interpolating the Ct values from the unknown samples against the corresponding developed standard curve and expressed as log_10_ CFU per gram of plant material or ml of grapevine must or wine.

#### 2.5.2 Dilution-plate counting

Plant material washing was 10-fold serially diluted, and appropriate dilutions were seeded by 20 μL drops onto agar plates. MRS agar supplemented with 50 μg ml^−1^ rifampicin (Sigma, Missouri, United States) and 50 μg ml^−1^ cycloheximide (Sigma) was used to counter-select PM411 and avoid fungal growth, respectively. To count presumptive A17, LB agar supplemented with 50 μg ml^−1^ cycloheximide (Sigma) was used, and samples were incubated at 80°C for 10 min before being seeded onto LB plates to inactivate vegetative cells and count spores. Plates were incubated at 28°C for 24–48 h. The culturable population levels of A17 and PM411 were expressed as the log_10_ CFU per gram of plant material.

### 2.6 Statistical analysis

A one-way analysis of variance (ANOVA) was performed to test the significance of (i) the effect of the quantification method to estimate the A17 and PM411 population levels on plant surfaces for each sampling time point and (ii) the performance of the viable population of the two mBCA on plant surfaces at each sampling time point. Means of the log_10_ of CFU g^−1^ were separated according to Tukey’s or Student’s *t*-test at a *p* value of ≤0.05 (IBM SPSS Statistics for Windows, version 25.0 released in 2017 by IBM Corp, Armonk, NY, United States). The lowest limit of detection (LDL) was used for statistical analysis when no amplification signal by qPCR and v-qPCR and no plate growth were obtained (3 log_10_ CFU g^−1^ for qPCR and v-qPCR; 2.7 log_10_ CFU g^−1^ for dilution-plate counting).

## 3 Results

**Figure 1:**
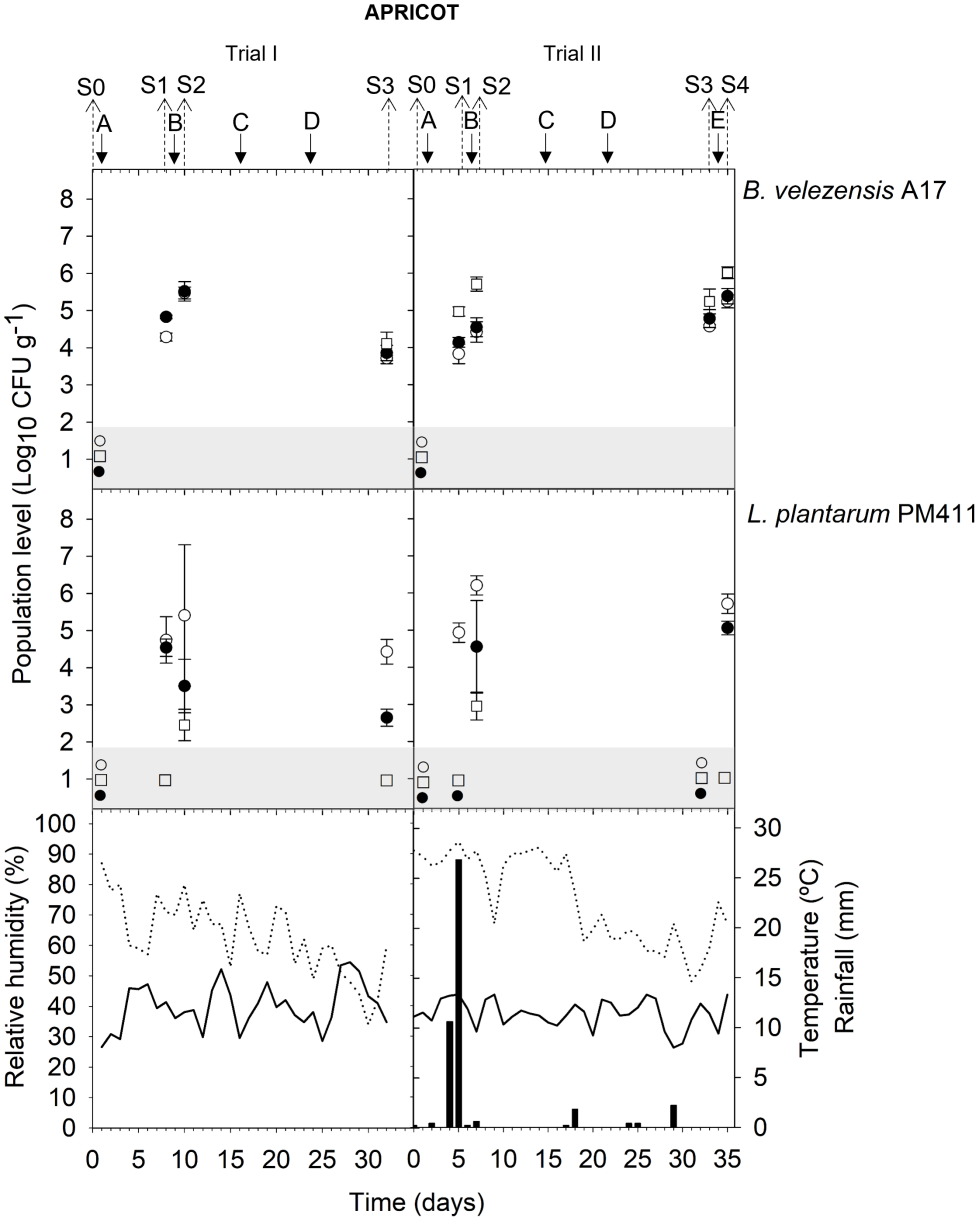
Tracking *B. velezensis* A17 and *L. plantarum* PM411 in apricot flowers (S0, S1, and S2) and immature fruits (S3, S4). Two field trials (I and II) were performed (Supplementary Table S1). Population levels estimated by qPCR (○), v-qPCR (●), and dilution-plate counting (□). Symbols placed in the grey zone mean below the limit of detection (2.7 Log_10_ CFU g^−1^ for dilution-plate counting and 3 Log_10_ CFU g^−1^ for qPCR and v-qPCR). No data available from dilution-plate counting at S1 and S2 from trial I for *B. velezensis* A17. Treatment applications are represented with solid arrows and capital letters **(A–E)**, while sampling time points (S) are represented with dotted arrows. Mean daily temperature (black line), amount of rainfall (black bars), and relative humidity (dotted line) were recorded during the trials.

**Figure 2:**
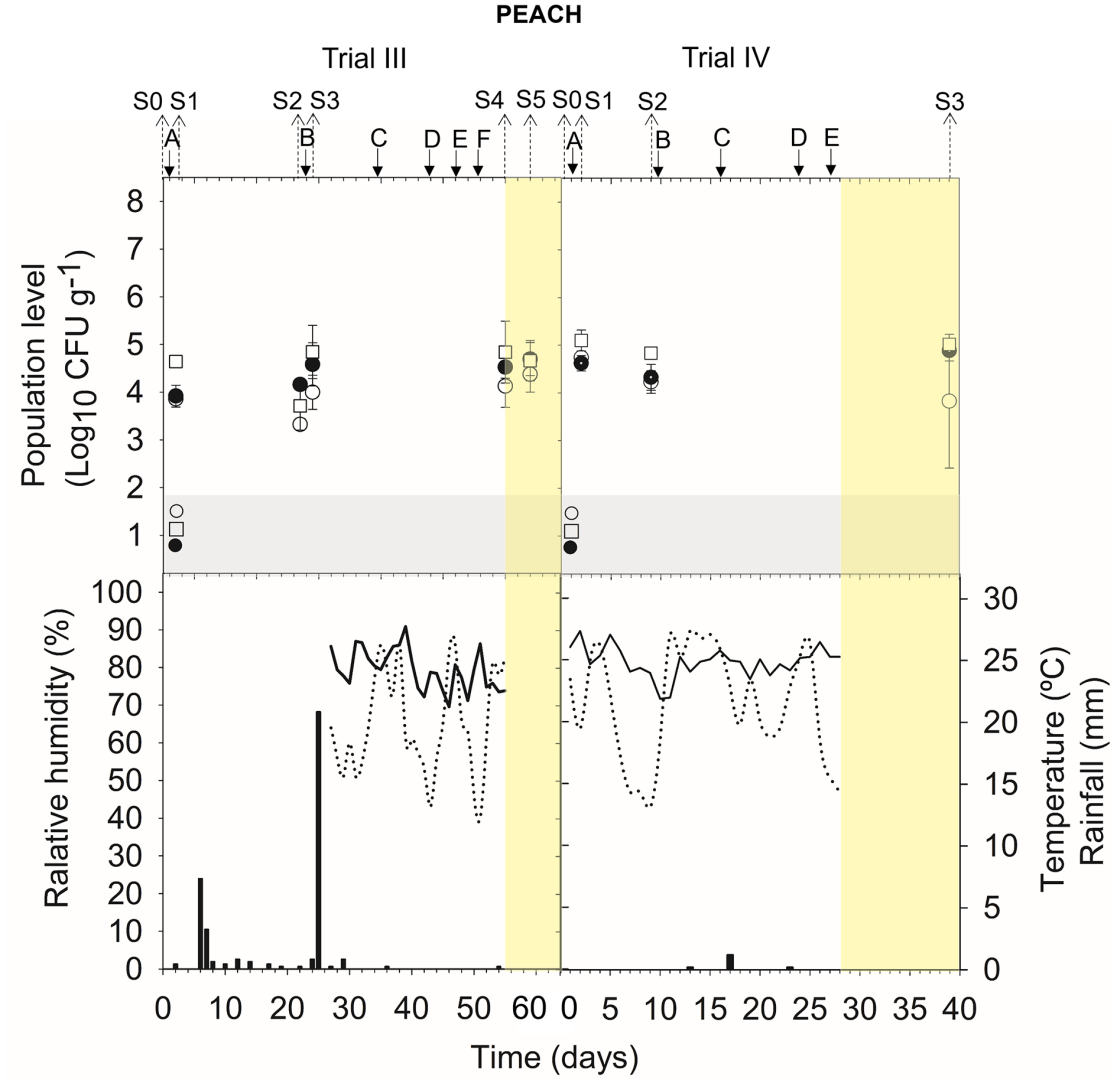
Tracking *B. velezensis* A17 in peach fruits throughout the treatments in the orchard (S0, S1, S2, and S3 in trial III and S0, S1, and S2 in trial IV) and during post-harvest (S4 and S5 in trial III and S3 in trial IV). Two field trials (III and IV) were performed (Supplementary Table S1). Population level of *B. velezensis* A17 estimated by qPCR (○), v-qPCR (●), and dilution-plate counting (□). Symbols placed in the grey zone mean below the limit of detection (2.7 Log_10_ CFU g^−1^ for dilution-plate counting and 3 Log_10_ CFU g^−1^ for qPCR and v-qPCR). Treatment applications are represented with solid arrows and capital letters **(A–F)**, while sampling time points (S) are represented with dotted arrows. Yellow zone means post-harvest samplings. Mean daily temperature (black line), amount of rainfall (black bars), and relative humidity (dotted line) were recorded during the trials.

**Figure 3:**
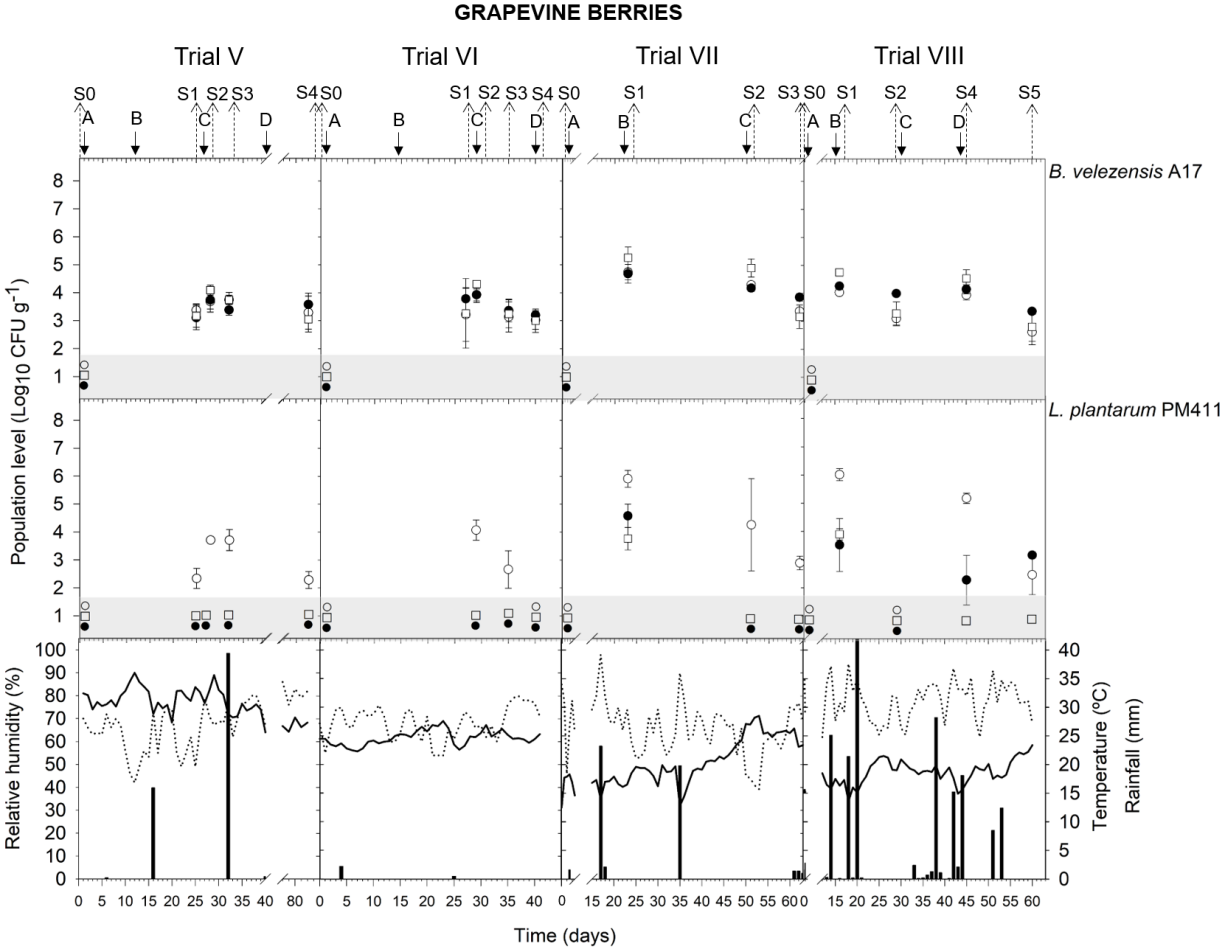
Tracking of *B. velezensis* A17 and *L. plantarum* PM411 in grapevine berries. Four field trials (V, VI, VII, and VIII) were performed (Supplementary Table S1). Population levels estimated by qPCR (○), v-qPCR (●), and dilution-plate counting (□). Symbols placed in the grey zone mean below the limit of detection (2.7 Log_10_ CFU g^−1^ for dilution-plate counting and 3 Log_10_ CFU g^−1^ for qPCR and v-qPCR). Treatment applications are represented with solid arrows and capital letters **(A–D)**, while sampling time points (S) are represented with dotted arrows. Mean daily temperature (black line), amount of rainfall (black bars), and relative humidity (dotted line) were recorded during the trials.

**Figure 4:**
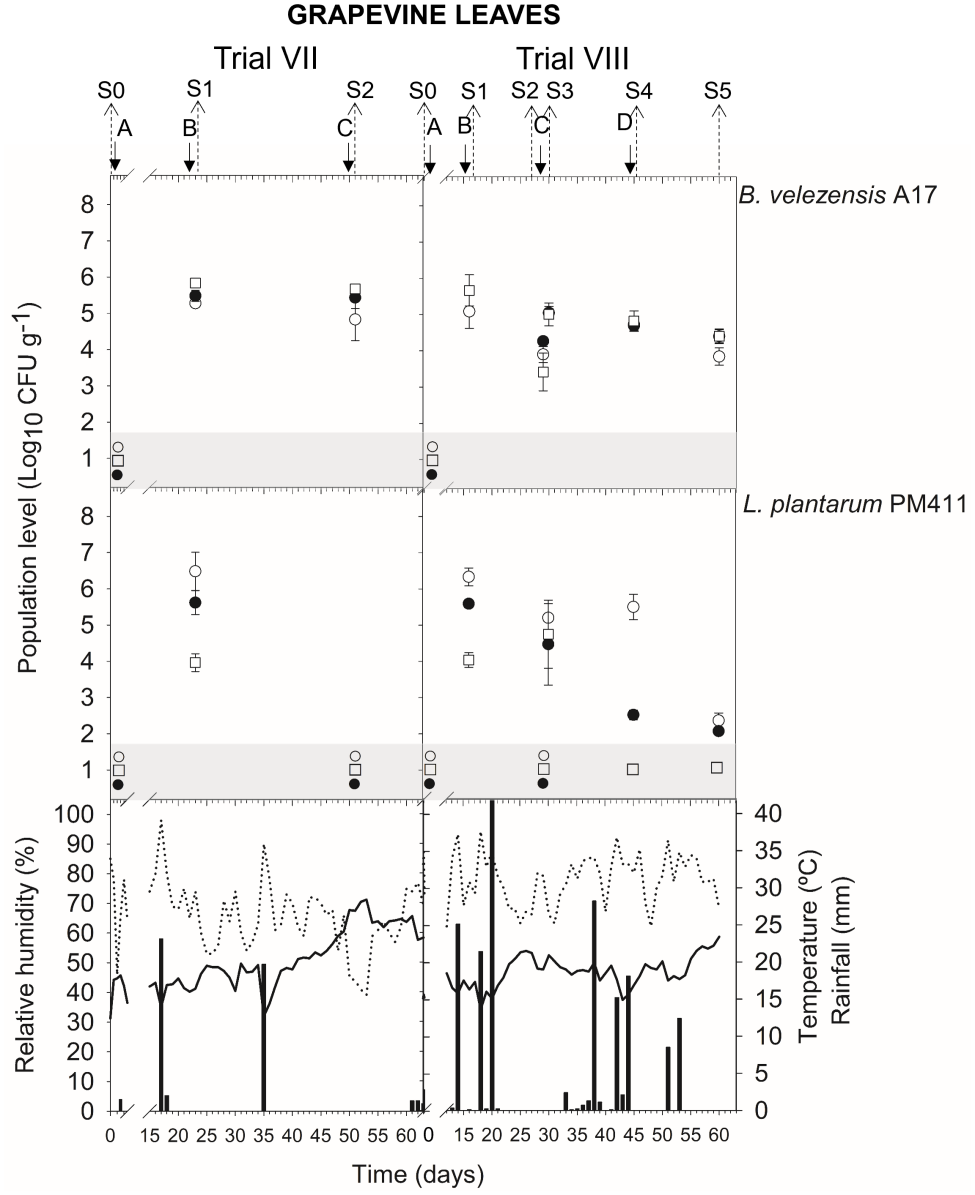
Tracking of *B. velezensis* A17 and *L. plantarum* PM411 in grapevine leaves. Two field trials (VII and VIII) were performed (Supplementary Table S1). Population levels estimated by qPCR (○), v-qPCR (●), and dilution-plate counting (□). Symbols placed in the grey zone mean below the limit of detection (2.7 Log_10_ CFU g^−1^ for dilution-plate counting and 3 Log_10_ CFU g^−1^ for qPCR and v-qPCR). Treatment applications are represented with solid arrows and capital letters **(A–D)**, while sampling time points (S) are represented with dotted arrows. Mean daily temperature (black line), amount of rainfall (black bars), and relative humidity (dotted line) were recorded during the trials.

**Figure 5:**
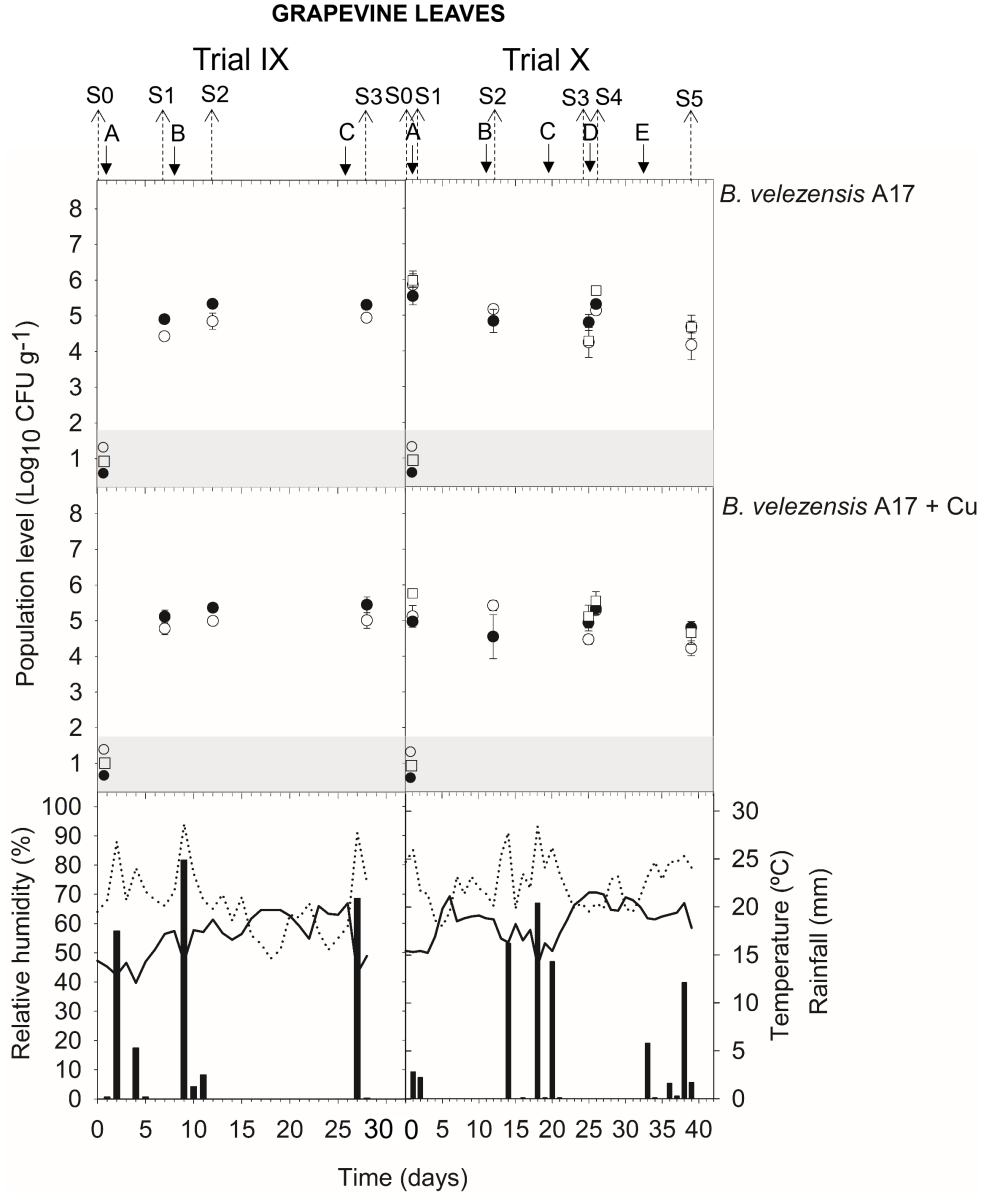
Tracking of *B. velezensis* A17 in grapevine leaves. Two field trials (IX and X) were performed (Supplementary Table S1). Population levels estimated by qPCR (○), v-qPCR (●), and dilution-plate counting (□). Symbols placed in the grey zone mean below the limit of detection (2.7 Log_10_ CFU g^−1^ for dilution-plate counting and 3 Log_10_ CFU g^−1^ for qPCR and v-qPCR). No data available from dilution-plate counting at S1, S2, and S3 from trial IX for *B. velezensis* A17 with and without Cu. Treatment applications are represented with solid arrows and capital letters **(A–E)**, while sampling time points (S) are represented with dotted arrows. Mean daily temperature (black line), amount of rainfall (black bars), and relative humidity (dotted line) were recorded during the trials.

Meteorological data (relative humidity, RH; mean daily temperature; and amount of rainfall) from apricot and peach orchards and vineyards are indicated in the corresponding figure of population dynamics (Figures 1–5) for the experimental period when treatment applications and samplings were carried out.

Neither *B. velezensis* A17 nor *L. plantarum* PM411 was detected by qPCR before the first inoculation (sampling time point S0) in any of the field trials and in grapevine must and wine (data not shown).

### 3.1 Population dynamics of *Bacillus velezensis* A17

The total population level of *B. velezensis* A17 in all plant organs, including apricot flowers and immature fruits, peach fruits, and grapevine leaves and berries, was viable (Figures 1–5). No significant differences were observed between qPCR and v-qPCR estimations in the most sampling time points in all trials (except for S1 trial I, S2 trial III, S2 trial IV, S5 trial VIII leaves, S1, S2, and S3 trial IX, S4 trial X without Cu, and S5 trial X with Cu) (Supplementary Table S5). In addition, the viable population level of A17 was also culturable since dilution-plate counting estimated similar values to v-qPCR in all trials. However, at some sampling time points, not only A17 spores were quantified by dilution-plate counting using LB as the growth medium but also spores from indigenous *Bacillus* spp. with similar morphology were counted. Significantly higher values by dilution-plate counting were estimated than qPCR and v-qPCR at several sampling time points (S2, and S4 trial II, S1 trial III, S2 trial VII berries, S1 trial VII leaves, S1 trial VIII berries, and S4 trial X without Cu) (Supplementary Table S5).

#### 3.1.1 Apricot (trials I and II)

The viability of A17 was estimated at approximately 4.5–5.6 log CFU g^−1^ both in flowers and immature fruits after 24 h of its application on trees (S2 trial I; S2 and S4 trial II) (Figure 1) (Supplementary Table S5). In addition, this level was stable at approximately 3.9–4.8 log CFU g^−1^ after 5 or 7 days of application in flowers (S1 trial I and trial II) and after 7 or 12 days in immature fruits (S3 trial I and trial II). Although rainfall was recorded between application A and sampling S1 in trial II, 4.1 log CFU g^−1^ of viable cells was quantified (Figure 1).

#### 3.1.2 Peach (trials III and IV)

The viability of A17 was estimated at approximately 3.9–4.8 log CFU g^−1^ after 24 h of its application on trees (S1 and S3 trial III, S1 trial IV) (Figure 2) (Supplementary Table S5). The level of viable cells was stable (4.1–4.9 log CFU g^−1^) for 9–20 days in the orchard (S2 in trial III and IV) and for 7–14 days post-harvest (S4 and S5 trial III; S3 trial IV).

#### 3.1.3 Grapevine (trials from V To X)

In berries, the viable population level was estimated at approximately 3.9–4.7 log CFU g^−1^ within 24 h after *B. velezensis* A17 application (S1 trial VII; S2 trial VI, VII; and S4 trial VIII) (Figure 3) (Supplementary Table S5). Throughout the following 6–16 days after application (S1 trial V and VI; S2 trial VIII; S3 trial V, VI, VII; and S5 trial VIII), the viable population was stable at 3.1–4.0 log CFU g^−1^. In addition, 3.6 log CFU g^−1^ was determined at harvest (S4 trial V).

Rainfall was recorded after treatment applications B and C in trial V and applications B and D in trial VIII (Figure 3), and the A17 viable population levels estimated on berries were 3.1–3.4 log CFU g^−1^ (S1 and S3 V) and 3.3–4.0 log CFU g^−1^ (S2 and S5 in trial VIII).

In leaves, the viable population level was estimated at approximately 4.6–5.5 log CFU g^−1^ within 24 h after *B. velezensis* A17 application (S1 trial VII and X; S2 trial VII and X; S3 trial VIII; and S4 trial VIII and X) (Figures 4, 5) (Supplementary Table S5). Throughout the next 4–7 days after application (S1 and S2 trial IX; S3 trial X), the viable population was stable at 4.9–5.3 log CFU g^−1^, whereas it decreased after 14–16 days (S2 and S5 trial VIII) until 4.2–4.4 log CFU g^−1^. Interestingly, the survival of *B. velezensis* A17 on grapevine leaves, as determined by v-qPCR when it was applied alone or in combination with copper, did not show significant differences (except in S1 trial X) (Supplementary Table S5).

Rainfall was recorded after treatment applications B and D in trial VIII, A, B, and C in trial IX, and C and E in trial X (Figures 4, 5), and the viable population levels of A17 estimated on leaves were approximately 4.2–4.4 log CFU g^−1^ (S2 and S5 trial VIII), 4.9–5.4 log CFU g^−1^ (S1, S2, and S3 in trial IX), and 4.7–4.9 log CFU g^−1^ (S3 and S5 in trial X) (Supplementary Table S5).

### 3.2 Population dynamics of *Lactiplantibacillus plantarum* PM411

The presence of dead, viable, and/or VBNC *L. plantarum* PM411 cells was confirmed on apricot and grapevine plant materials because significant differences between dilution-plate counting, qPCR, and v-qPCR estimations were observed (except for S3 trial VI, S2 trial VII berries, S3 leaves, and S5 leaves and berries trial VIII) (Figures 1, 3, 4) (Supplementary Table S6). Furthermore, qPCR showed higher values than v-qPCR, and v-qPCR showed higher values than dilution-plate counting.

#### 3.2.1 Apricot (trials I and II)

The total population level of PM411 after 24 h of its application on trees (S2 trial I; S2 and S4 trial II) was 5.7–6.2 log CFU g^−1^, both in flowers and immature fruits according to qPCR estimations (Figure 1) (Supplementary Table S6). Only a portion of the total population level was viable, specifically between 0.2 and 22.5% since v-qPCR estimated approximately 3.5–5.1 log CFU g^−1^. In addition, the presence of VBNC PM411 cells was confirmed because dilution-plate counting estimations were below 3.0 log CFU g^−1^. Between 0.0 and 0.1% of the total population of PM411 were viable and culturable cells.

In addition, the PM411 viable population decreased in 5–12 days (S3 trial I and S1 and S3 trial II) below the v-qPCR limit of quantification or detection (3 log CFU g^−1^), despite being estimated at approximately 4.4–4.9 log CFU g^−1^ by qPCR in S3 trial I and S1 trail II, indicating the presence of dead PM411 cells (Figure 1) (Supplementary Table S6).

#### 3.2.2 Grapevine (trials from V To VIII)

In berries, PM411 showed different patterns depending on the field trial (Figure 3) (Supplementary Table S6). In trials V and VI (samplings in July and August), PM411 did not survive, and only dead cells were detected in berries. While the quantification by qPCR was below 4 log CFU g^−1^, viable PM411 was neither detected by v-qPCR nor dilution-plate counting. In trials VII and VIII (samplings in May and June), the total population level of PM411 on berries was 4.3–6.0 log CFU g^−1^ after 24 h of application (S1 and S2 trial VII, S1 and S4 trial VIII), according to qPCR estimations. Only a portion of the total population level was viable, specifically between 0 and 4.7%, since v-qPCR estimated approximately 2.3–4.6 log CFU g^−1^ or did not detect PM411. In addition, the presence of VBNC PM411 cells was confirmed because dilution-plate counting estimations were below 3.7 log CFU g^−1^. The total population of PM411 was viable and culturable Between 0.0 and 0.7% of the total population of PM411 were viable and culturable cells. At other sampling time points, viable PM411 was either not detected or quantified under the lowest quantification level (3 log CFU g^−1^) by v-qPCR.

In leaves, within 24 h after application of B (S1 trial VII and VIII), the total population levels of PM411 were estimated approximately 6.3–6.5 log CFU g^−1^ by qPCR (Figure 4) (Supplementary Table S6). In total, 13–20% of the PM411 population level was viable since approximately 5.6 log CFU g^−1^ was quantified by v-qPCR, whereas 0.3–0.5% was viable and culturable since approximately 4.0 log CFU g^−1^ was quantified by dilution-plate counting.

When the field performance of A17 and PM411 is compared, no differences were observed between strains in the viable population level after 24 h of application in apricot (S2 and S4 in trial II) and grapevine (S1 in trial VII leaves and berries, S1 berries, and S3 leaves in trial VIII). However, the viability of PM411 was significantly lower than A17 after 5–16 days of application in both crops (S3 trial I; S1 and S3 trial II; S2 and S3 trial VII; S2, S4, and S5 trial VIII) (Supplementary Tables S7–S9).

## 4 Discussion

In the present study, the assessment of population dynamics of *B. velezensis* A17 and *L. plantarum* PM411, after their application to three plant crops and two geographical zones over two growing seasons, was studied by comparing qPCR, v-qPCR, and dilution-plate counting estimations. Therefore, the population level of dead, viable, and VBNC cells could be differentiated. Following the recommendation, a polyphasic approach, combining classical and molecular techniques, is interesting to evaluate the dynamics of any inoculant strain after its release into the environment to know its survival and performance (Manfredini et al., 2021). The maintenance of higher densities of mBCAs for at least part or even the whole growing season is an important property in making them effective (Köhl et al., 2019).

*Bacillus velezensis* A17 and *L. plantarum* PM411 showed different dynamics of foliar and fruit colonization and persistence. Specifically, *B. velezensis* A17 showed higher survival than *L. plantarum* PM411 in preharvest applications in the field, both in apricot trees and vineyards. The different behavior between strains can be explained since, among the microorganisms, there are strains that can be considered as generalists, colonizing a wide range of host plants, whereas others are more restricted (Sessitsch et al., 2019). For example, *Pseudomonas fluorescens* EPS62e was previously reported as an mBCA that was able to reach very high population levels on apple and pear blossoms in the orchard both in Atlantic and Mediterranean climatic conditions. In addition, its colonization pattern on blossoms was not greatly influenced by the host plant species (Pujol et al., 2007). In the case of *L. plantarum* PM411, a previous study reported that while stable population levels of viable cells were attained in the flower environment under high relative humidity, the unfavorable conditions on the leaf surface and the relatively dry conditions in the field caused an important decrease in the viable population (Daranas et al., 2018b).

However, for both strains, the estimation of the viable population level on grapevine berries was approximately 1 log less than on grapevine leaves. This phenomenon may be explained by different features of the surfaces of leaves and berries. On the one hand, the composition of the bacterial communities has been reported to be significantly different (Leveau and Tech, 2011). For example, the surface of grapevine berries is highly colonized by yeasts (Barata et al., 2012). On the other hand, the leaf cuticle layer plays a critical role in mediating interactions with microorganisms, and several structures present on leaves provide colonization sites for them (Chaudhry et al., 2021).

The total population level of *B. velezensis* A17, after field application in apricot, peach, and grapevine, was viable. This result demonstrated that the qPCR approach was suitable for monitoring the A17 strain. The usefulness of qPCR, as a monitoring tool of mBCA after delivery on plants, was also confirmed in *P. fluorescence* EPS62e, which showed efficient colonization of blossoms (Pujol et al., 2006, 2007). However, higher population levels of viable and culturable A17 were estimated by non-selective dilution-plate counting using LB agar. This result is consistent with the fact that other spores from indigenous bacteria could grow in the medium with similar morphology to A17. However, contrary to our results, a previous report demonstrated that the dilution-plate counting methodology using the non-selective nutrient yeast glucose (NYDA) agar was suitable for monitoring the *B. amyloliquefaciens* CPA-8 strain, primarily as a consequence of the characteristic colony morphology, which makes it difficult to confuse CPA-8 with the other microbiota (Vilanova et al., 2018).

Interestingly, *B. velezensis* A17 showed high survival rates in peach fruits, apricot flowers, immature fruits, and grapevine leaves and berries during the whole period of the study after field inoculations, regardless of the date when spray applications were performed. *B. velezensis* A17 was subjected to different climate conditions, mainly temperature, depending on the field trial. Specifically, apricot trees were treated in February and March (trials I and II) and, on average, 11–12°C of temperature and 63–75% of relative humidity were recorded. In the case of peach trees, they were treated in July and August (trials III and IV) and, on average, 24–25°C of temperature and 65–70% of relative humidity were recorded. While grapevines were treated from April to June (trials VII, VIII, IX, and X) with an average temperature of 17–19°C and relative humidity of 67–79%, they were also treated as well as from July to August (trials V and VI) with an average temperature of 25–30°C and relative humidity of 67_68%. In addition, when rainfall occurred throughout the days after treatment application, A17 attained stable population levels of viable cells at approximately 3–4 log CFU g^−1^ (S1 trial II, S1 and S3 trial V, and S2 and S5 trial VIII) and 4–5 log CFU g^−1^ (S1, S2, and S3 trial IX and S3 and S5 trial X) for the following days. Therefore, rainfall did not wash off A17 populations on apricot flowers or grapevine berries and leaves. As it was reported in other studies, allowing populations of mBCA to establish for 24 h or more, prior to a rain event, significantly increases persistence on grape berries, and the effect of rain intensity is not observable (Calvo-Garrido et al., 2014).

High survival rates of A17 in the field are consistent with those reported by other authors monitoring *B. amyloliquefaciens* CPA-8 biocontrol strain, that highlighted its ability to survive largely on leaves and fruit surfaces after preharvest application in the field (Gotor-Vila et al., 2017; Vilanova et al., 2018; Casals et al., 2021). These results are consistent with the fact that the spore-forming ability of *Bacillus* species provides high resistance to field environmental conditions (Mendis et al., 2018), making this genus a good candidate for developing stable and efficient biocontrol products. In addition, despite subsequent applications of the A17 strain, the carrying capacities of apricot flowers and immature fruits (5.5 log CFU g^−1^), grapevine berries and leaves (3.7–4.7 and 5.5 log CFU g^−1^, respectively), and peach fruits (4.8 log CFU g^−1^) were limited by the resources available for growth (Wilson and Lindow, 1992).

Interestingly, the simultaneous application of *B. velezensis* A17 and copper did not have a negative effect on A17 survival on grapevine leaves. Taking into account that, in organic vineyards, the use of Cu-based pesticides is currently the only chemical treatment allowed, limited to a maximum of 6 kg Cu ha^−1^ per year in the European Union (Gobbi et al., 2020), the combined application of A17 and copper is considered to achieve acceptable disease control, reducing the amount of Cu-based pesticides used. According to these results, *B. velezensis* A17 could establish and survive actively over a broad range of crops and climatic conditions.

On the contrary, the total population level of *L. plantarum* PM411 in apricot and grapevine was made up of dead, culturable, and/or VBNC cells since significant differences were observed between the three methods. Therefore, under harsh conditions, the presence of dead and VBNC cells was confirmed since higher population levels of PM411 were determined by qPCR, followed by v-qPCR and plate-dilution plating using selective media MRS amended with rifampicin. This finding was also reported in other studies conducted with the biocontrol strain *P. agglomerans* CPA-2 applied on citrus fruits under field conditions (Soto-Muñoz et al., 2015a).

The studies conducted in other bacteria confirmed the persistence of DNA during several weeks after cell death and the promotion of cells to enter into a VBNC state due to stressful conditions as a strategy of survival and adaptation to the field environment (Pinto et al., 2015; Soto-Muñoz et al., 2015a; Papadimitriou et al., 2016). Although qPCR approach was previously reported as the monitoring tool for the evaluation of population dynamics of the biocontrol strain *L. plantarum* MW-1 applied on grapevine leaves in the field (Gobbi et al., 2020), in the present study, it was demonstrated that the viability of qPCR approach was the most accurate for the assessment of viable population dynamics of *L. plantarum* PM411.

The cell viability of *L. plantarum* PM411 drastically declined over time after application in apricot trees and vineyards. In particular, PM411 survives on grapevine berries and leaves only within 24 h after applications, which were carried out during May and June (trials VII and VIII) (average temperature of 19°C, average RH of 67–76%) but not during July and August (trials V and VI) (average temperature of 25–30°C, average RH of 67–68%). Therefore, *L. plantarum* PM411 did not survive on grapevine berries during summertime. In fact, in a previous study in which PM411 was applied to apple and pear blossoms in the field during springtime environmental conditions, a decreased survival was observed (Daranas et al., 2018b). The population dynamics of PM411 on grapevine leaves in the field are consistent with the previously reported survival of the biocontrol strain *L. plantarum* BY on leaves of Chinese cabbage in the field (Tsuda et al., 2016). As reported in other mBCAs, such as *P. agglomerans* CPA-2, populations rapidly declined in chambers with low RH contents (Cañamás et al., 2008), as well as on citrus fruits in the field (Soto-Muñoz et al., 2015a). These observations are consistent with the hypothesis that, in the phyllosphere, *L. plantarum* PM411 is exposed to stress environmental factors, such as light intensity, low RH, wind, UV radiation exposition and high temperature, scarce nutrient availability, and high redox potential, which negatively affect its establishment (Cañamás et al., 2008; Sui et al., 2015; Lamont et al., 2017).

Higher populations of PM411 viable cells were estimated at 24 h after application in apricot flowers and immature fruits (Figure 1) than in grapevine berries (Figure 3) and leaves (Figures 4, 5). The flower environment is favorable for bacterial survival and colonization because of a high level of nutrients. As reported in a previous study, PM411 efficiently colonized pear and apple flowers (Roselló et al., 2013). On the contrary, the leaf environment is poor in carbon-containing nutrients and more exposed to fluctuations in temperature, UV radiation, and especially water availability (Lindow and Brandl, 2003; Redford et al., 2010).

These results highlight the importance of improving *L. plantarum* PM411 formulation to achieve better survival on different crops and climate conditions (Daranas et al., 2018a).

In conclusion, using culturable and qPCR-based methods (conventional and viability) for strain monitoring, we have demonstrated that *B. velezensis* A17 and *L. plantarum* PM411 differ strongly in their colonization and survival in grapevine and apricot as well as in their response to environmental conditions. While A17 attained a high survival rate regardless of the trial, the survival capacity of PM411 was highly constrained by the crop and climate conditions. Therefore, the development of strain-specific molecular methods aims to quantify the viable population level of an mBCA, which is required to manage its field applications and achieve acceptable biocontrol of diseases. The combined use of these methods could provide potential tools for future studies on their spread and fitness in orchards, as well as their influence on the native microbiome.

## Data availability statement

The datasets presented in this study can be found in online repositories. The names of the repository/repositories and accession number(s) can be found at: https://www.ncbi.nlm.nih.gov/genbank/, NZ_JRYS01000000 and MG788324.

## Author contributions

ND: Conceptualization, Methodology, Writing – review & editing, Data curation, Formal analysis, Investigation, Writing – original draft. EB: Conceptualization, Methodology, Writing – review & editing, Funding acquisition, Supervision. EM: Conceptualization, Funding acquisition, Methodology, Supervision, Writing – review & editing. AB: Conceptualization, Funding acquisition, Methodology, Supervision, Writing – review & editing.
