# Supplementary material for: Colonization and population dynamics of total, viable, and culturable cells of two biological control strains applied to apricot, peach, and grapevine crops
Source: Front Microbiol. 2024 Jan 5;14:1324965. doi: 10.3389/fmicb.2023.1324965 (PMC10797078; doi:10.3389/fmicb.2023.1324965)
Supplement: Supplementary file 1 [file Data_Sheet_1.docx]

Supplementary Material

**Development of quantitative PCR (qPCR) and viability qPCR (v-qPCR)**

The strain-specific qPCR and v-qPCR for *B. velezensis* A17 were developed in the present study. For *L. plantarum* PM411, strain-specific qPCR and v-qPCR were previously reported (Daranas et al. 2018). However, in the present work, the v-qPCR was updated using the PMAxx® reagent as the nucleic acid-binding dye.

The strain-specific qPCR and v-qPCR approaches enabled the detection and tracking of the total and viable population level of A17 and PM411 strains when released into orchards of apricot and peach, and in vineyards.

# Materials and methods

## Identification of a *B. velezensis* A17 strain-specific molecular marker

Genome sequencing analysis was carried out to identify *B. velezensis* A17 strain-specific molecular markers. *B. velezensis* A17 genomic DNA was sequenced using the Illumina MiSeq platform (250 bp paired-end reads). Genome comparative analysis between A17 (NZ_JRYS01000000) and Y2 (GenBank accession number CP003332.1) strains was carried out using Mauve (Darling et al., 2004) and Artemis (Rutherford et al., 2000) software. The specificity of the putative unique regions at strain level were ensured *in silico* using the BLAST program at NCBI database (http://www.ncbi.nlm.nih.gov/BLAST/).

Then, one PCR assay was developed per each putative specific region. Oligonucleotide sequences, amplification mixtures and PCR conditions are described in Table I and Table II. A non-template control (NTC), using water instead of genomic DNA, and a positive control with A17 DNA were included in all PCR runs. DNA from QST713, FZB42, and A59 *Bacillus* strains were also used (Table III) and obtained according to the method described by Llop et al. (1999) with some modifications (Mora et al., 2011).

The amplification products were separated by electrophoresis on a 1.8 % (w/v) agarose gel in 1× Tris-acetate Disodium Ethylenediaminetetraacetate dehydrate (EDTA) and stained with ethidium bromide. Size comparisons were made with a 1-kb plus ladder (Invitrogen, California, USA). Gel images were captured with an imaging system (Kodak 120; Kodak, Rochester, NY, USA).

**Table I.** Oligonucleotides designed for PCR assays to test *B. velezensis* A17 strain-specificity of 8 putative unique regions.

| **PCR assay** | **Oligonucleotide** | **Sequence (5’-3’)** | **Amplicon length (bp)** |
| --- | --- | --- | --- |
| R.1 | Forward | CGCTGATGTCGTAACGCTTG | 415 |
|  | Reverse | ATTTTCCCAGGCGATCAGCA |  |
| R.2 | Forward | ATACGCCGGGTTTTAAGCGA | 751 |
|  | Reverse | GCGATCGCTCTCGACTATGG |  |
| R.3 | Forward | TTCTCCGGGAAAGAGATTGCC | 267 |
|  | Reverse | TCTGAATTCCACACTGCCCA |  |
| R.4 | Forward | TGACGACGAAAGTTGGGACAA | 847 |
|  | Reverse | TCCTTTTCGAAAGCACTGGC |  |
| R.5 | Forward | ACTCTTCCTCGATTGCTCCTT | 744 |
|  | Reverse | TCAGCTTGAGTACGGGGGTA |  |
| R.6 | Forward | GCTAACGGCGTCAGTGAAAC | 552 |
|  | Reverse | ACCTAACCAGAAAGCCACGG |  |
| R.7 | Forward | GGTGAGCGGGAATAGAAGGC | 481 |
|  | Reverse | TTGTCAGCAGGAATCGGAATTG |  |
| R.8 | Forward | GTGCTCCTTCACCGGTATCC | 619 |
|  | Reverse | AGCATTTTCAGTGCTTTTTCAACT |  |

**Table II.** Amplification mixture and PCR conditions to test *B. velezensis* A17 strain-specificity of 8 putative unique regions.

| **Amplification mixture** | **PCR conditions ^c^** |
| --- | --- |
| 1x PCR buffer ^a^, 2 mM MgCl_2_ ^a^, 0.2 mM dNTP ^a^, 1U Taq DNA Polymerase ^a^, 0.2 μM Forward primer ^b^, 0.2 μM Reverse primer ^b^, Mili-Q H_2_O and 2 µL genomic DNA (10 ng μL-^1^) (reaction volume 25 µL) | 95ºC 5 min, 40 cycles of (95ºC for 30 s, 58ºC for 30 s, and 72ºC 1 min), 72ºC for 1 min, and 4ºC for ∞ |
| ^a^ Invitrogen  ^b^ Table I  ^c^ PCR was carried out in a T3000 thermocycler (Biometra, Germany) | |

**Table III.** Bacterial strains of *Bacillus* species used in this study.

| ***Bacillus* species** | **Code strain** |
| --- | --- |
| *B. velezensis* | A17^a^, FZB42^b^, UMAF6639^c^, QST713^d^ |
| *B. amyloliquefaciens* | 5 strains isolated from plant sources^a^ (A59, A63, A132, A135, A164) |
| *B. subtilis* | 23 strains isolated from plant sources^a^ (A1, A2, A5, A7, A9, A11, A12, A13, A16, A20, A22, A23, A24, A29, A31, A55, A68, A87, A88, A112, A113, A118, A119) |
| *B. pumilus* | 7 strains isolated from plant sources^a^ (A14, A25, A26, A28, A68, A77, A84) |
| *B. megaterium* | 2 strains isolated from plant sources^a^ (A19, A130) |
| *B. cereus* | 3 strains isolated from plant sources^a^ (A37, A38, A73) |
| *B. thuringensis* | 1 strain isolated from plant sources^a^ (A83) |
| *B. licheniformis* | 3 strains isolated from plant sources^a^ (A3, A32, A151) |
| ^a^ CIDSAV, University of Girona, Spain (Mora et al., 2011, 2015)  ^b^ isolated from RhizoVital^®^ 42, ABiTEP GmbH, Germany  ^c^ Department of Microbiology, University of Malaga, Spain.  ^d^ isolated from Serenade Max^®^, Bayer Cropscience, Germany | |

## Design of *B. velezensis* A17 strain-specific TanMan-based qPCR assay

Three TaqMan-based qPCR assays were designed within the best putative unique region found in *B. velezensis* A17 genome to obtain three amplicons with different lengths (144 bp, A; 277 bp, B; 323 bp, C). Although the optimal amplicon length for qPCR assays to guarantee method efficiency is less than 100 bp (Bustin et al., 2009), in v-qPCR, longer DNA sequences are necessary (Martin et al., 2013; Daranas et al., 2018) considering the influence of amplicon length in the effectiveness of the PMAxx treatment to suppress PCR amplification of dead cells.

Primers and TaqMan probes used for qPCR analysis are listed in Table IV. Probes were labelled with the 6-carboxyfluoresceine (FAM) reporter dye at 5’ end and with the 6-carboxytetramethylrhodamine (TAMRA) quencher dye at 3’ end. Primers and TaqMan probes designs were carried out using the Primer Express 3.0 software (Applied Biosystem, 380 Foster City, USA). The specificity of the TaqMan qPCR designs for A17 strain was tested using a collection of 47 *Bacillus* strains belonging to six species of the genus (Table III) after optimization of the concentration of the primers and probes. NTC, using water instead of genomic DNA, and positive control with A17 DNA were included in all qPCR runs. All reactions were performed in triplicate and were carried out in a QuantStudio 5 Real-time PCR system (Applied Biosystem, Foster City, USA). Amplification mixture and qPCR conditions are described in Table V.

**Table IV.** Primers and TaqMan probes used for qPCR analysis to amplify *B. velezensis* A17 and *L. plantarum* PM411.

| **Primer/probe** | **Sequence (5’-3’)** | **Amplicon length (bp)** | **Reference** |
| --- | --- | --- | --- |
| ***B. velezensis* A17** | | | |
| A17-F1 | CGCGAACAAACGTTCTTGGT | 144 (A) | This work |
| A17-R1 | TCAGCTTGAGTACGGGGGTA |  |  |
| A17-P1 ^b^ | TGGTACAAGTTGGGAAAGCAATTGGT |  |  |
|  |  |  |  |
| A17-F2 | TTAAACCAGCAAGGACAAATTCAC | 277 ^a^ (B) | This work |
| A17-F3 | CCATTGATGATTCTCGACTTCACT | 323 ^a^ (C) | This work |
| A17-R3 | CAGGAATTCCCCTATTTTTGTTGA |  |  |
| A17-P3 ^b^ | TTAACATCGCGGAAAAAGAAGTCAGCATCC |  |  |
| ***L. plantarum* PM411** | |  |  |
| PM411-For | AGATGCCAGCACTGGATTAAGC | 188 | Daranas et al. (2018) |
| PM411B-Rev | CCTTGTCGATACCAAAGTTAGCTATG |  |  |
| PM411-pr | TGCACGGCACAACTCAGGCGATT |  |  |
| ^a^ Amplicon sizes are amplification products obtained by qPCR using the corresponding forward primer (A17-F2 or A17-F3), A17-R3 reverse primer and A17-3P TaqMan probe.  ^b^ Probes were labelled with the 6-carboxyfluoresceine (FAM) reporter dye at 5’ end and with the 6-carboxytetramethylrhodamine (TAMRA) quencher dye at 3’ end. | | | |

**Table V.** Amplification mixture and qPCR conditions to amplify *B. velezensis* A17 and *L. plantarum* PM411.

| **qPCR assay** | **Amplification mixture ^a^** | **qPCR conditions ^b^** |
| --- | --- | --- |
| A17 | 1x TaqMan Universal PCR Master Mix, 300 nM each forward and reverse primer, 250 nM of probe, and 20 ng DNA or 2 µL DNA sample (reaction volume 25 µL). | 50ºC 2 min, 95ºC 10 min, 50 cycles of (95ºC for 15 s and 60ºC 1 min). |
| PM411 | 1x TaqMan Universal PCR Master Mix, 500 nM each forward and reverse primer, 250 nM of probe, and 20 ng DNA or 4 µL DNA sample (reaction volume 20 µL). |  |
| ^a^ TaqMan Universal PCR master mix is manufactured by Invitrogen.  ^b^ qPCR was carried out in a QuantStudio 5 Real-time PCR system (Applied Biosystems). | | |

## PMAxx concentration optimization for *B. velezensis* A17 and *L. plantarum* PM411 v-qPCR assays

PMAxx reagent (Biotium, Fremont, California, USA) was diluted in 900 μl of sterile diethyl pyrocarbonate (DEPC)-treated water (Ambion™, Austin, Texas, USA) to obtain a stock solution of 2000 μM that was stored at -20ºC in the dark until needed. To determine the optimal concentration of PMAxx (20, 25 and 50 μM) an appropriate volume of PMAxx stock solution (10, 12.5 or 25 μl) was added into 1 mL of viable or dead vegetative cell suspension of A17 or PM411 strains at 5 x 10^6^ CFU mL^-1^ in water. A17 vegetative cells were harvested from a 3-h-old liquid culture at log-phase, where sporulation was not initiated, while PM411 cells were harvested from a 24-h-old solid culture. Dead cells were obtained by heating a cell suspension at 100ºC for 30 min (ThermoMixer F1.5; Eppendorf). The loss of viability of vegetative cell was checked by plating on LB or MRS agar followed by incubation for 24-48h at 28ºC. Then, cell suspensions were thoroughly mixed and incubated for 10 min in the dark at room temperature in an orbital shaker KS501 digital (IKA Labortechnik) at 130 rpm. Immediately, samples were photo-activated for 15 min with the PhAST Blue photoactivation system (GenIUL, Barcelona, Spain) set to 100% and transferred into DNA low binding 1.5 mL tubes (Sarstedt, Nümbrecht, Germany). PMAxx treated cells (viable and dead) were collected by centrifugation at 13200xg for 10 min and washed with 50 mM sterile PBS (pH 7.0) under the same centrifugation conditions. Samples of viable and dead cells without being treated with PMAxx were also used. DNA extraction of PMAxx and non-PMAxx treated samples was performed according to the method described by (Schmidt et al., 2008) with some modifications (Daranas et al., 2018). The qPCR runs for A17 (144 bp, A) and PM411 were performed in triplicate as described above in section 1.2. The effect of PMAxx on DNA amplification suppression was tested in viable and dead cells and expressed as “signal reduction”. Signal reduction was calculated by subtracting Ct values between non-PMAxx and PMAxx-treated samples.

## Standard curves for v-qPCR quantification of A17 and PM411 viable cells

To check the v-qPCR method as a strain-specific bacterial detection and quantification tool of viable cells, the sensitivity and amplification efficiency of the v-qPCR assays for A17, and PM411 were evaluated by developing standard curves.

Cell suspensions of viable or dead vegetative cells of A17 and PM411 were prepared in sterile distilled water at high concentration (10^8^ CFU mL^-1^) and diluted to appropriate concentrations with plant-material washing (peach peal and grapevine leaves). The cell concentration was checked by OD_600_ measure, considering that 0.6 and 0.3 corresponds to 10^8^ CFU mL^-1^ for *Bacillus* and *Lactiplantibacillus*, respectively, and was confirmed by colony counts. The tested concentrations covered a 5-log range, from 1 x 10^2^ to 1 x 10^7^ CFU mL^-1^. An aliquot of plant-material washings without inoculating A17 and PM411 cells was kept as NTC sample. To obtain plant-material washings, peach fruits (‘Corindon’) from experimental orchards near Torreilles (France) and grapevine leaves (‘Macabeu’) from a commercial orchard near Vilafranca del Penedés (Spain) were used. Plant material (2-5 g) was infused with 50 mM sterile phosphate buffer (PBS, pH 7.0) and 0.1% peptone (at the proportion of 1g / 10 mL) in a 100 mL sterile bottle and mixed in an orbital shaker (KS501 digital; IKA Labortechnik, Staufen, Germany) at 130 rpm for 30 min on ice. From each sample, 1 mL was treated with PMAxx at 20 μM and DNA was isolated following the procedure described above.

The strain-specific TaqMan qPCR assays for A17 (144 bp, A) and PM411 were performed, and two NTC were included in all PCR runs: (i) using water instead of genomic DNA, and (ii) using DNA isolated from plant-material washings without A17 or PM411 cells. All reactions were performed in triplicate as described above in section 1.2. Ct values were plotted against the logarithm of the initial number of CFU mL^-1^ and standard curves were generated by a linear regression of the plotted points. Slopes were used to determine the amplification efficiency of each design from the equation E (%) = (10 ^-1/slope^ – 1) x 100.

## Statistical analysis

A one-way analysis of variance (ANOVA) was performed to test the significance of the effect of PMAxx concentration in the suppression of DNA amplification (signal reduction) on dead and viable cells of A17, and PM411. Means of the ∆Ct (signal reduction) were separated according to the Tukey’s test at a *p* value of ≤0.05 (IBM SPSS Statistics for Windows, Version 25.0 released on 2017 by IBM Corp, Armonk, NY, United States).

## Accession number

The genome sequence of *B. velezensis* A17 was deposited in the GenBank database under the accession number NZ_JRYS01000000. The putative phage sequence of *L. plantarum* PM411, where the PM411-molecular marker is located, was deposited in the GenBank database under the accession number MG788324.

# Results

## Strain-specific TaqMan-based qPCR

Eight putative unique regions were identified *in silico* in *B. velezensis* A17 genome. One of them (Region 5) proved strain-specificity since no amplifications by the *Bacillus* strains QST713, FZB42, and A59 were confirmed (Figure I).


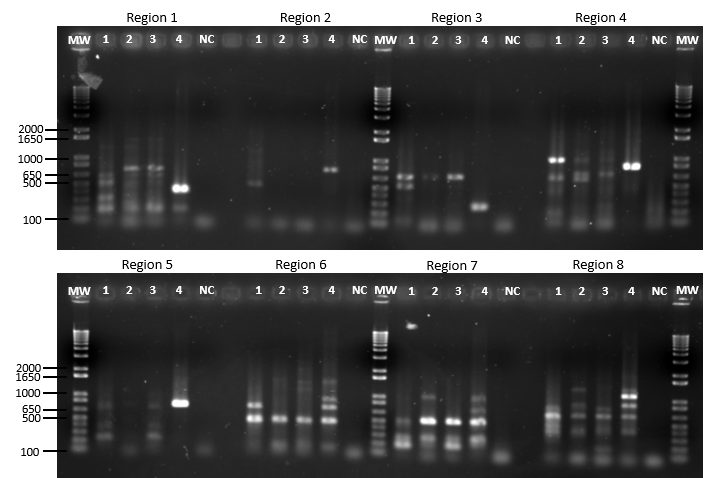


**Figure I.** PCR analysis of the eight putative *B. velezensis* A17 strain-specific regions. *Bacillus* strains: QST713 (1), FZB42 (2), A59 (3), and A17 (4). NC, negative control for each PCR assay. MW, 1Kb Plus DNA Ladder (Invitrogen).

The strain-specific molecular marker ‘Region 5’ was located in “NODE_2_length_673145_cov_63.100425” from A17 genome (GenBank accession number JRYS02000002). This 744 bp-length sequence is partially annotated by RASTk pipeline as a hypothetical protein. The *in-silico* analysis using BLAST to align the complete genome of A17 and the 744 bp-length sequence revealed that this is a single-copy sequence (data not shown).

Three strain-specific TaqMan-based qPCR assays were developed within the molecular marker and successful amplifications of A17 DNA were achieved with Ct values of 22-24 (A, 144 bp), 26-28 (B, 277 bp), and 30-32 (C, 323 bp) (Table IV). Considering the sensitivity of the three A17 qPCR assays, only the A17-A was used for further studies. The 144 bp-length sequence amplified was not annotated by RASTk pipeline. The *in-silico* analysis using BLAST to align the complete genome of A17 and the 144 bp-length sequence revealed that this is a single-copy sequence.

No amplification or random fluorescence signals at Ct values higher than 35 was observed with the 93.6% of *Bacillus* strains listed in Table III. Only three *Bacillus* strains from UdG-CIDSAV collection, specifically A130, A151, and A164 strains, were amplified by the three TaqMan-based qPCR assays with similar Ct values to A17. However, these strains are probably siblings of A17 because all they were isolated in the same location during the same screening stage in Estartit (Catalonia, Spain) despite of coming from different samples.

The specificity of the PM411-strain specific molecular marker with TaqMan qPCR assay was previously reported (Daranas et al., 2018).

## Viability qPCR

The effect of different PMAxx concentrations on the amplification of DNA targets of viable and dead cells was studied. The signal reduction value (SR) for each qPCR design, defined as the difference of Ct value between non-PMAxx-treated and PMAxx-treated samples (∆Ct) was determined (Figure II). No significant differences of SR between concentrations of PMAxx were observed neither on dead or viable cells in each strain. 20 μM PMAxx was the lowest concentration showing good results as inhibited the DNA amplification of A17 and PM411 dead cells at 1 x 10^6^ CFU mL^-1^, while viable cells were not affected. Therefore, 20 μM was chosen for further experiments.


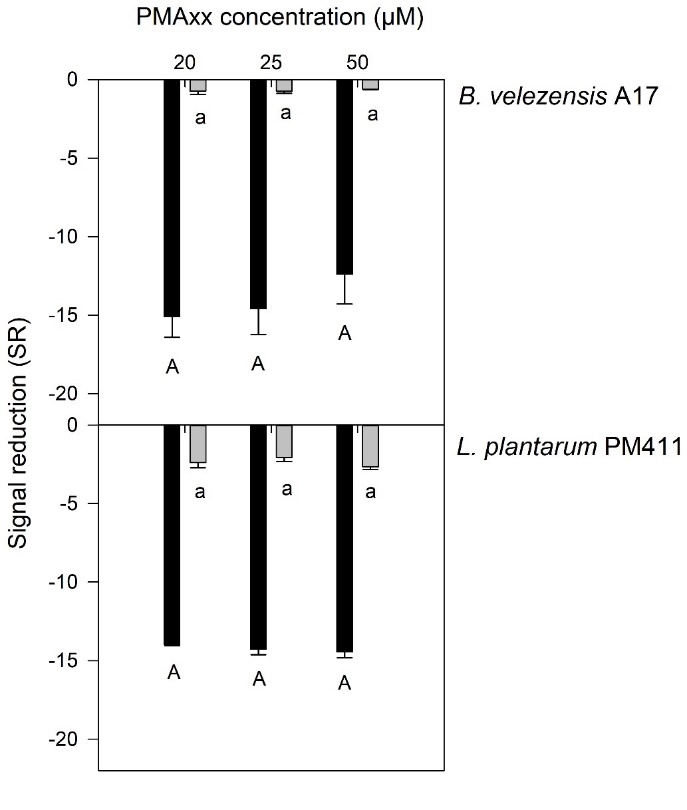


**Figure II.** Signal reduction (SR) of the amplification by qPCR using different PMAxx concentrations on viable (grey bars) and dead (black bars) cells of *B. velezensis* A17, and *L. plantarum* PM411. SR is the difference between cycle threshold values (ΔCt) of non-PMAxx and PMAxx-treated samples. The results are shown as means from three independent replicates and error bars represent standard deviations of the mean. Different letters (capital in dead and lowercase in viable cell suspensions) in the same panel indicate significant differences between concentrations of PMAxx reagent (P < 0.05) according to the Tukey test.

Standard curves were developed in plant-material washings (peach peal and grapevine leaves) using the two strain-specific assays (A17 and PM411) combined with PMAxx treatment (Figure III). Viable cells showed high correlation coefficient values (R^2^ = 0.98-0.99) and amplification efficiencies above 88% (Table VI). The relationship was linear over the range of 1 x 10^2^ to 1 x 10^7^ CFU mL^-1^. On dead cells treated with PMAxx, Ct values higher than 38 were obtained over the range from 1 x 10^2^ to 1 x 10^5^ CFU mL^-1^, meaning that the amplification was completely inhibited. Only, at concentrations of 1 x 10^6^ and 1 x 10^7^ CFU mL^-1^ (and 1 x 10^5^ CFU mL^-1^ for PM411) lower Ct values were reached, namely higher than 28, and 25 for A17, and PM411, respectively. The detection and quantification limits of the v-qPCR were 3 log CFU g^-1^, regardless of the sample matrix (Table VI).


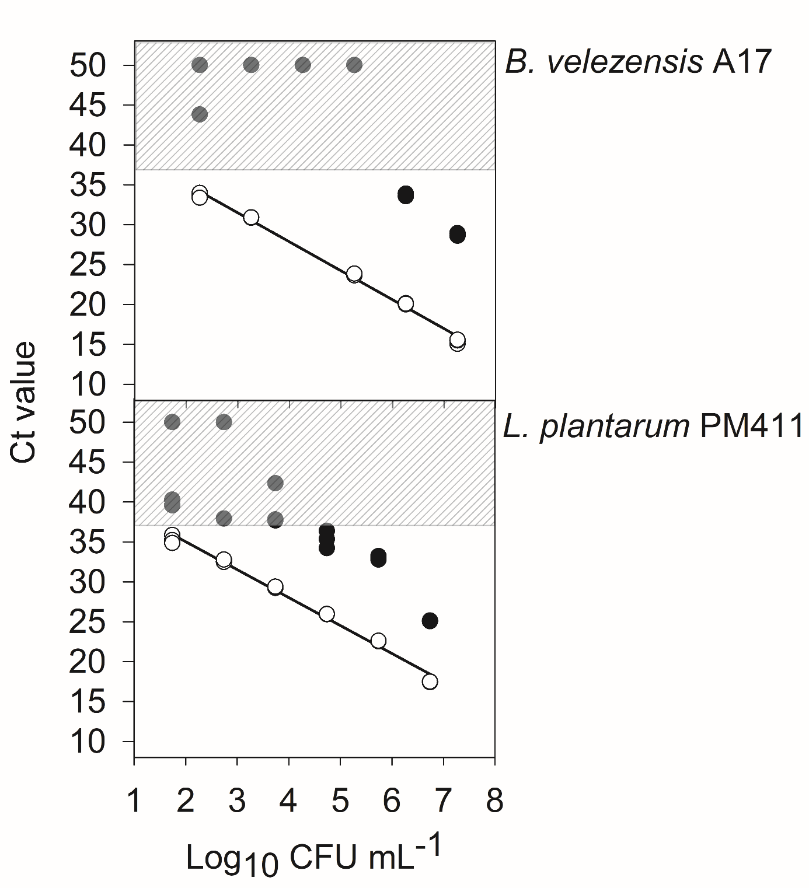


**Figure III.** Standard curves of *B. velezensis* A17 in peach peal, and *L. plantarum* PM411 in grapevine leaves using the strain-specific assays A17, and PM411, respectively, combined with PMAxx treatment (v-qPCR). White dots mean viable cells and black dots mean dead cells. Three replicates (three dots) per each standard point. The striped background represents the detection limit at Ct values of >38.

**Table VI.** Parameters of the regression curves for the calibration of the viability qPCR vs. viable cell concentration for *B. velezensis* A17, and *L. plantarum* PM411 strains.

| **v-qPCR assay** | **Plant material matrix** | **Slope (a)** | **Intersection (b)** | **R^2^** | **LOG Range (CFU mL^-1^)** | **Sensitivity ^a^**  **(Ct value)** | **Efficiency ^b^ (%)** |
| --- | --- | --- | --- | --- | --- | --- | --- |
| *B. velezensis* A17 | Peach peal washing | -3.626 | 42.348 | 0.994 | 2.3 – 7.3 | 33.6 (1.8 x 10^2^) | 88.69 |
| *L. plantarum* PM411 | Grapevine leaves washing | -3.503 | 42.050 | 0.989 | 1.7 – 6.7 | 35.3 (5 x 10^1^) | 92.97 |
| ^a^ Ct value at the lowest quantification level (CFU mL^-1^)  ^b^ Equation: Ct = -a · Log_10_ CFU·mL^-1^ + b | | | | | | | |

# Acknowledgements

The authors would like to thank Jordi Cabrefiga for the molecular marker identification of A17 strain by bioinformatic analysis, and Francis Cazorla from Department of Microbiology, University of Malaga for providing UMAF6639 strain.

# References

Bustin, S. A., Benes, V., Garson, J. A., Hellemans, J., Huggett, J., Kubista, M., et al. (2009). The MIQE guidelines: minimum information for publication of quantitative real-time PCR experiments. *Clin Chem* 55, 611–622. doi: 10.1373/clinchem.2008.112797.

Daranas, N., Bonaterra, A., Francés, J., Cabrefiga, J., Montesinos, E., and Badosa, E. (2018). Monitoring viable cells of the biological control agent *Lactobacillus plantarum* PM411 in aerial plant surfaces by means of a strain-specific viability quantitative PCR method. *Appl Environ Microbiol* 84, e00107-18. doi: 10.1128/AEM.00107-18.

Darling, A. C. E., Mau, B., Blattner, F. R., and Perna, N. T. (2004). Mauve: multiple alignment of conserved genomic sequence with rearrangements. *Methods* 14, 1394–1403.

Llop, P., Caruso, P., Cubero, J., Morente, C., and López, M. M. (1999). A simple extraction procedure for efficient routine detection of pathogenic bacteria in plant material by polymerase chain reaction. *J Microbiol Methods* 37, 23–31. doi: 10.1016/S0167-7012(99)00033-0.

Martin, B., Raurich, S., Garriga, M., and Aymerich, T. (2013). Effect of amplicon length in propidium monoazide quantitative PCR for the enumeration of viable cells of *Salmonella* in cooked ham. *Food Anal Methods* 6, 683–690. doi: 10.1007/s12161-012-9460-0.

Mora, I., Cabrefiga, J., and Montesinos, E. (2011). Antimicrobial peptide genes in *Bacillus* strains from plant environments. *International microbiology* 14, 213–223. doi: 10.2436/20.1501.01.151.

Mora, I., Cabrefiga, J., and Montesinos, E. (2015). Cyclic lipopeptide biosynthetic genes and products, and inhibitory activity of plant-associated *Bacillus* against phytopathogenic bacteria. *PLoS One* 10, e0127738. doi: 10.1371/journal.pone.0127738.

Rutherford, K., Parkhill, J., Crook, J., Horsnell, T., Rice, P., Rajandream, M.-A., et al. (2000). Artemis: sequence visualization and annotation. *Bioinformatics* 16, 944–945. doi: 10.1093/bioinformatics/16.10.944.

Schmidt, R. J. J., Emara, M. G. G., Kung Jr, L., and Kung, L. (2008). The use of a quantitative real-time polymerase chain reaction assay for identification and enumeration of *Lactobacillus buchneri* in silage. *J Appl Microbiol* 105, 920–9. doi: 10.1111/j.1365-2672.2008.03834.x.
