# Supplementary material for: Colonization and population dynamics of total, viable, and culturable cells of two biological control strains applied to apricot, peach, and grapevine crops
Source: Front Microbiol. 2024 Jan 5;14:1324965. doi: 10.3389/fmicb.2023.1324965 (PMC10797078; doi:10.3389/fmicb.2023.1324965)
Supplement: Supplementary file 2 [file Data_Sheet_2.docx]

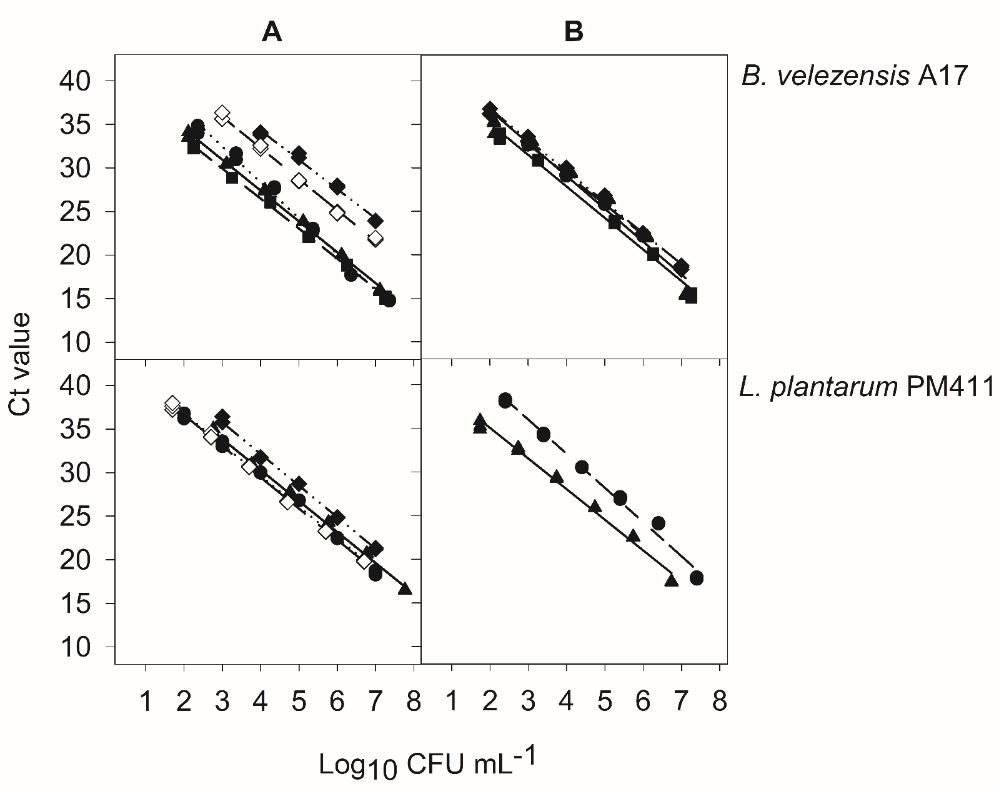


**Supplementary Figure S1.** Standard curves of *B. velezensis* A17, and *L. plantarum* PM411 TaqMan qPCR (A) and viability qPCR (B) assays in different plant material washings (apricot flowers, ●; peach peal, ■; grapevine leaves, ▲), grapevine must, ♦ and wine, ◊. Three replicates (three symbols) per each standard point.

**Supplementary Table S1.** Field trials and schedule of treatment applications and samplings.

| **Trial code** | **Crop** | **Location** |  | **Applications ^a^** | **BBCH scale ^b^** | **Dates of application ^c^** | **Sampling time points (S)** |
| --- | --- | --- | --- | --- | --- | --- | --- |
| I | Apricot | Torreilles (France) |  | A  B  C  D | 57-59  65  67-69  72 | 25/02/2019  05/03/2019  12/03/2019  21/03/2019 | S0: 1 d before A  S1: 1 d before B  S2: 1 d after B  S3: 7 d after D |
| II |  |  |  | A  B  C  D  E | 57  57  59  65  71 | 18/02/2021  24/02/2021  04/03/2021  11/03/2021  24/03/2021 | S0: 1 d before A  S1: 1 d before B  S2: 1 d after B  S3: 1 d before E  S4: 1 d after E |
| III | Peach | Torreilles (France) |  | A  B  C  D  E  F | 75  75  77  81  83  87 | 03/07/2019  25/07/2019  05/08/2019  14/08/2019  19/08/2019  23/08/2019 | S0: 1 d before A  S1: 1 d after A  S2: 1 d before B  S3: 1 d after B  S4: 3 d after F (harvest)  S5: 7 d after F (postharvest) |
| IV |  |  |  | A  B  C  D  E | 76  81  83  85  87 | 28/07/2020  06/08/2020  10/08/2020  18/08/2020  21/08/2020 | S0: 1 d before A  S1: 1 d after A  S2: 1 d before B  S3: 1 d after B  S4: 14 d after E (postharvest) |
| V | Grapevine | Sant Martí Sarroca (Spain) |  | A  B  C  D | 75-77  75-77  79  81 | 12/07/2019  23/07/2019  06/08/2019  20/08/2019 | S0: 1 d before A  S1: 1 d before C  S2: 2 d after C  S3: 6 d after C  S4: 42 d after D (harvest) |
| VI |  | Vilafranca del Penedès (Spain) |  | A  B  C  D | 75-77  79  79  81 | 09/07/2020  23/07/2020  06/08/2020  19/08/2020 | S0: 1 d before A  S1: 1 d before C  S2: 1 d after C  S3: 7 d after C  S4: 1 d before D |
| VII |  | La Granada (Spain) |  | A Sulphur  B  C  D Sulphur | 53  57  73-75  77 | 08/05/2019  30/05/2019  26/06/2019  10/07/2019 | S0: 1 d before A  S1: 1 h after B  S2: 1 d after C  S3: 12 d after C |
| VIII |  |  |  | A  B  C  D | 53  57  65  73 | 27/04/2020  12/05/2020  26/05/2020  09/06/2020 | S0: 1 d before A  S1: 1 h after B  S2: 1 h before C  S3: 1 h after C  S4: 1 d after D  S5: 16 d after D |
| IX |  | Gelida (Spain) |  | A  B  C | 53  57  65-69 | 16/05/2019  23/05/2019  10/06/2019 | S0: 1 d before A  S1: 6 d after A  S2: 4 d after B  S3: 2 d after C |
| X |  |  |  | A  B  C  D  E | 53  55  60  65  69-75 | 27/04/2020  08/05/2020  15/05/2020  22/05/2020  28/05/2020 | S0: 1 d before A  S1: 1 h after A  S2: 1 h after B  S3: 7 d after C  S4: 1 h after D  S5: 7 d after E |
| ^a^ Capital letters mean the applications carried out in each trial.  ^b^ BBCH scale define the phenological development stage of the crop when applications were performed (Meier 2018).  ^c^ Dates of application are presented as day/month/year. | | | | | | | |

**Supplementary Table S2.** Primers and TaqMan probes used for strain-specific qPCR and v-qPCR analysis to amplify *B. velezensis* A17 and *L. plantarum* PM411.

| **Primer/probe** | **Sequence (5’-3’)** | **Amplicon length (bp)** | **Reference** |
| --- | --- | --- | --- |
| ***B. velezensis* A17** | | | |
| A17-F1 | CGCGAACAAACGTTCTTGGT | 144 | This work  (supplementary material) |
| A17-R1 | TCAGCTTGAGTACGGGGGTA |  |  |
| A17-P1 ^a^ | TGGTACAAGTTGGGAAAGCAATTGGT |  |  |
|  |  |  |  |
| ***L. plantarum* PM411** | |  |  |
| PM411-For | AGATGCCAGCACTGGATTAAGC | 188 | Daranas *et al.* (2018b) |
| PM411B-Rev | CCTTGTCGATACCAAAGTTAGCTATG |  |  |
| PM411-pr ^a^ | TGCACGGCACAACTCAGGCGATT |  |  |
| ^a^ Probes were labelled with the 6-carboxyfluoresceine (FAM) reporter dye at 5’ end and with the 6-carboxytetramethylrhodamine (TAMRA) quencher dye at 3’ end. | | | |

**Supplementary Table S3**. Amplification mixture and qPCR conditions to amplify *B. velezensis* A17 and *L. plantarum* PM411.

| **qPCR assay** | **Amplification mixture ^a^** | **qPCR conditions ^b^** |
| --- | --- | --- |
| A17 | 1x TaqMan Universal PCR Master Mix, 300 nM each forward and reverse primer, 250 nM of probe, and 20 ng DNA or 2 µL DNA sample (reaction volume 25 µL). | 50ºC 2 min, 95ºC 10 min, 50 cycles of (95ºC for 15 s and 60ºC 1 min). |
| PM411 | 1x TaqMan Universal PCR Master Mix, 500 nM each forward and reverse primer, 250 nM of probe, and 20 ng DNA or 4 µL DNA sample (reaction volume 20 µL). |  |
| ^a^ TaqMan Universal PCR master mix is manufactured by Invitrogen.  ^b^ qPCR was carried out in a QuantStudio 5 Real-time PCR system (Applied Biosystems). | | |

**Supplementary Table S4.** Parameters of the regression curves for the calibration of the qPCR vs. cell concentration for *B. velezensis* A17, and *L. plantarum* PM411 strains.

| **Assay** | **Plant material matrix** | **Slope (a)** | **Intersection (b)** | **R^2^** | **Log_10_ Range (CFU mL^-1^)** | **Sensitivity ^a^**  **(Ct value)** | **Efficiency ^b^ (%)** |
| --- | --- | --- | --- | --- | --- | --- | --- |
| **qPCR** |  |  |  |  |  |  |  |
| *B. velezensis* A17 | Apricot flower washing | -4.120 | 44.841 | 0.992 | 2.4 – 7.4 | 34.4 (2.3 x 10^2^) | 74.87 |
|  | Peach peal washing | -3.444 | 40.277 | 0.998 | 2.3 – 7.3 | 32.4 (1.8 x 10^2^) | 95.15 |
|  | Grapevine leaves washing | -3.539 | 41.563 | 0.996 | 2.1 – 7.1 | 33.7 (1.3 x 10^2^) | 91.68 |
|  | Must | -3.359 | 47.740 | 0.990 | 4 - 7 | 33.9 (1 x 10^4^) | 98.47 |
|  | Wine | -3.569 | 46.593 | 0.996 | 3 - 7 | 36.0 (1 x 10^3^) | 90.61 |
|  |  |  |  |  |  |  |  |
| *L. plantarum* PM411 | Apricot flower washing | -3.849 | 43.737 | 0.992 | 1.7 – 6.7 | 34.6 (5 x 10^1^) | 81.89 |
|  | Grapevine leaves washing | -3.554 | 40.866 | 0.998 | 2 – 7 | 36.4 (1 x 10^2^) | 91.15 |
|  | Must | -3.625 | 46.640 | 0.996 | 3 - 7 | 36.1 (1 x 10^3^) | 88.75 |
|  | Wine | -3.586 | 43.790 | 0.999 | 1.7 - 6.7 | 37.6 (5 x 10^1^) | 90.50 |
|  |  |  |  |  |  |  |  |
| **v-qPCR** |  |  |  |  |  |  |  |
| *B. velezensis* A17 | Apricot flower washing | -3.469 | 43.144 | 0.999 | 3 – 6 | 32.8 (1 x 10^3^) | 94.19 |
|  | Peach peal washing | -3.626 | 42.348 | 0.994 | 2.3 – 7.3 | 33.6 (1.8 x 10^2^) | 88.69 |
|  | Grapevine leaves washing | -3.783 | 44.223 | 0.961 | 2.1 – 7.1 | 34.8 (1.3 x 10^2^) | 83.81 |
|  |  |  |  |  |  |  |  |
| *L. plantarum* PM411 | Apricot flowers washing | -3.914 | 47.730 | 0.991 | 2.4 – 7.4 | 38.1 (3 x 10^2^) | 80.16 |
|  | Grapevine leaves washing | -3.503 | 42.050 | 0.989 | 1.7 – 6.7 | 35.3 (5 x 10^1^) | 92.97 |
| ^a^ Ct value at the lowest quantification level (CFU mL^-1^)  ^b^ Equation: Ct = -a · Log_10_ CFU·mL^-1^ + b | | | | | | | |

**Supplementary Table S5**. Statistical analysis of population dynamics (Log_10_ CFU g^-1^) of *B. velezensis* A17 estimated by qPCR, v-qPCR and dilution plate counting (PC) at different sampling time points.

|  |  |  |  | **Sampling time point** | | | | | | | | | | | | | | | | | | |
| --- | --- | --- | --- | --- | --- | --- | --- | --- | --- | --- | --- | --- | --- | --- | --- | --- | --- | --- | --- | --- | --- | --- |
|  |  |  |  | **S1** | | |  | **S2** | | |  | **S3** | | |  | **S4** | | |  | **S5** | | |
|  | **Trial ^a^** | **Quantification method** |  | Mean^b^ | SD^c^ | *p* value^d^ |  | mean | SD | *p* value |  | mean | SD | *p* value |  | mean | SD | *p* value |  | mean | SD | *p* value |
| **Apricot** | **I** |  |  |  |  |  |  |  |  |  |  |  |  |  |  |  |  |  |  |  |  |  |
|  |  | qPCR |  | 4.29 | 0.11 | b |  | 5.48 | 0.16 |  |  | 3.72 | 0.15 |  |  | nd | - | -  - |  | nd | - | - |
|  |  | v-qPCR |  | 4.83 | 0.05 | a |  | 5.52 | 0.26 |  |  | 3.86 | 0.21 |  |  | nd | - |  |  | nd | - | - |
|  |  | PC |  | nd | - | - |  | nd | - | - |  | 4.11 | 0.31 |  |  | nd | - | - |  | nd | - | - |
|  |  |  |  |  |  | <0.001 |  |  |  | 0.392 |  |  |  | 0.101 |  |  |  | - |  |  |  | - |
|  | **II** |  |  |  |  |  |  |  |  |  |  |  |  |  |  |  |  |  |  |  |  |  |
|  |  | qPCR |  | 3.83 | 0.26 | a |  | 4.42 | 0.28 | b |  | 4.57 | 0.18 | b |  | 5.24 | 0.18 | b |  | nd | - | - |
|  |  | v-qPCR |  | 4.14 | 0.13 | a |  | 4.54 | 0.25 | b |  | 4.78 | 0.21 | ab |  | 5.38 | 0.21 | b |  | nd | - | - |
|  |  | PC |  | 1.97 | 0.12 | b |  | 5.71 | 0.19 | a |  | 5.24 | 0.16 | a |  | 6.01 | 0.16 | a |  | nd | - | - |
|  |  |  |  |  |  | <0.001 |  |  |  | <0.001 |  |  |  | 0.026 |  |  |  | 0.001 |  |  |  | - |
| **Peach** | **III** |  |  |  |  |  |  |  |  |  |  |  |  |  |  |  |  |  |  |  |  |  |
|  |  | qPCR |  | 3.86 | 0.17 | b |  | 3.32 | 0.12 | b |  | 4.00 | 0.36 |  |  | 4.13 | 0.45 |  |  | 4.37 | 0.37 |  |
|  |  | v-qPCR |  | 3.92 | 0.23 | b |  | 4.16 | 0.12 | a |  | 4.58 | 0.45 |  |  | 4.52 | 0.231 |  |  | 4.69 | 0.34 |  |
|  |  | PC |  | 4.64 | 0.13 | a |  | 3.71 | 0.51 | ab |  | 4.84 | 0.56 |  |  | 4.84 | 0.65 |  |  | 4.66 | 0.42 |  |
|  |  |  |  |  |  | 0.003 |  |  |  | 0.044 |  |  |  | 0.152 |  |  |  | 0.260 |  |  |  | 0.561 |
|  | **IV** |  |  |  |  |  |  |  |  |  |  |  |  |  |  |  |  |  |  |  |  |  |
|  |  | qPCR |  | 4.74 | 0.24 |  |  | 4.23 | 0.24 | b |  | 3.83 | 1.40 |  |  | nd | - | - |  | nd | - | - |
|  |  | v-qPCR |  | 4.62 | 0.16 |  |  | 4.32 | 0.27 | a |  | 4.89 | 0.22 |  |  | nd | - | - |  | nd | - | - |
|  |  | PC |  | 5.10 | 0.22 |  |  | 4.83 | 0.11 | a |  | 5.02 | 0.10 |  |  | nd | - | - |  | nd | - | - |
|  |  |  |  |  |  | 0.157 |  |  |  | 0.029 |  |  |  | 0.090 |  |  |  | - |  |  |  | - |
| **Grapevine** | **V** |  |  |  |  |  |  |  |  |  |  |  |  |  |  |  |  |  |  |  |  |  |
|  | Berries | qPCR |  | 3.37 | 0.23 |  |  | 3.68 | 0.38 |  |  | 3.74 | 0.16 |  |  | 3.28 | 0.59 |  |  | nd | - | - |
|  |  | v-qPCR |  | 3.10 | 0.43 |  |  | 3.74 | 0.32 |  |  | 3.38 | 0.19 |  |  | 3.57 | 0.41 |  |  | nd | - | - |
|  |  | PC |  | 3.16 | 0.40 |  |  | 4.09 | 0.17 |  |  | 3.65 | 0.25 |  |  | 3.26 | 0.17 |  |  | nd | - | - |
|  |  |  |  |  |  | 0.666 |  |  |  | 0.326 |  |  |  | 0.161 |  |  |  | 0.630 |  |  |  | - |
|  | **VI** |  |  |  |  |  |  |  |  |  |  |  |  |  |  |  |  |  |  |  |  |  |
|  | Berries | qPCR |  | 3.22 | 0.96 |  |  | 3.93 | 0.22 |  |  | 3.13 | 0.54 |  |  | 3.02 | 0.33 |  |  | nd | - | - |
|  |  | v-qPCR |  | 3.78 | 0.38 |  |  | 3.93 | 0.28 |  |  | 3.36 | 0.41 |  |  | 3.20 | 0.17 |  |  | nd | - | - |
|  |  | PC |  | 3.26 | 1.24 |  |  | 4.31 | 0.12 |  |  | 3.25 | 0.50 |  |  | 3.00 | 0.42 |  |  | nd | - | - |
|  |  |  |  |  |  | 0.725 |  |  |  | 0.117 |  |  |  | 0.847 |  |  |  | 0.723 |  |  |  | - |
|  | **VII** |  |  |  |  |  |  |  |  |  |  |  |  |  |  |  |  |  |  |  |  |  |
|  | Leaves | qPCR |  | 5.27 | 0.08 | b |  | 4.83 | 0.59 | b |  | nd | - | - |  | nd | - | - |  | nd | - | - |
|  |  | v-qPCR |  | 5.48 | 0.16 | b |  | 5.43 | 0.30 | ab |  | nd | - | - |  | nd | - | - |  | nd | - | - |
|  |  | PC |  | 5.83 | 0.08 | a |  | 5.67 | 0.10 | a |  | nd | - | - |  | nd | - | - |  | nd | - | - |
|  |  |  |  |  |  | <0.001 |  |  |  | 0.012 |  |  |  | - |  |  |  | - |  |  |  | - |
|  |  |  |  |  |  |  |  |  |  |  |  |  |  |  |  |  |  |  |  |  |  |  |
|  | Berries | qPCR |  | 4.74 | 0.28 |  |  | 4.28 | 0.34 | b |  | 3.34 | 0.38 | ab |  | nd | - | - |  | nd | - | - |
|  |  | v-qPCR |  | 4.68 | 0.03 |  |  | 4.17 | 0.12 | b |  | 3.84 | 0.33 | a |  | nd | - | - |  | nd | - | - |
|  |  | PC |  | 5.25 | 0.08 |  |  | 4.88 | 0.14 | a |  | 3.14 | 0.42 | b |  | nd | - | - |  | nd | - | - |
|  |  |  |  |  |  | 0.072 |  |  |  | <0.001 |  |  |  | 0.010 |  |  |  | - |  |  |  | - |
|  | continue | | | | | | | | | | | | | | | | | | | | | |

**Supplementary Table S5**. (continued)

|  |  |  |  | **Sampling time point** | | | | | | | | | | | | | | | | | | |
| --- | --- | --- | --- | --- | --- | --- | --- | --- | --- | --- | --- | --- | --- | --- | --- | --- | --- | --- | --- | --- | --- | --- |
|  |  |  |  | **S1** | | |  | **S2** | | |  | **S3** | | |  | **S4** | | |  | **S5** | | |
|  | **Trial ^a^** | **Quantification method** |  | Mean^b^ | SD^c^ | *p* value^d^ |  | mean | SD | *p* value |  | mean | SD | *p* value |  | mean | SD | *p* value |  | mean | SD | *p* value |
| **Grapevine** | **VIII** |  |  |  |  |  |  |  |  |  |  |  |  |  |  |  |  |  |  |  |  |  |
|  | Leaves | qPCR |  | 5.08 | 0.47 |  |  | 3.90 | 0.22 | ab |  | 5.05 | 0.12 |  |  | 4.72 | 0.08 |  |  | 3.84 | 0.24 | b |
|  |  | v-qPCR |  | nd | - | - |  | 4.27 | 0.07 | a |  | 5.05 | 0.18 |  |  | 4.69 | 0.14 |  |  | 4.40 | 0.20 | a |
|  |  | PC |  | 5.67 | 0.43 |  |  | 3.42 | 0.52 | b |  | 5.01 | 0.32 |  |  | 4.82 | 0.28 |  |  | 4.40 | 0.18 | a |
|  |  |  |  |  |  | 0.093 |  |  |  | 0.052 |  |  |  | 0.965 |  |  |  | 0.701 |  |  |  | 0.026 |
|  |  |  |  |  |  |  |  |  |  |  |  |  |  |  |  |  |  |  |  |  |  |  |
|  | Berries | qPCR |  | 4.03 | 0.09 | b |  | 3.10 | 0.25 |  |  | nd | - | - |  | 3.93 | 0.18 | b |  | 2.61 | 0.32 |  |
|  |  | v-qPCR |  | 4.26 | 0.09 | b |  | 3.99 | 0.01 |  |  | nd | - | - |  | 4.14 | 0.16 | ab |  | 3.35 | 0.11 |  |
|  |  | PC |  | 4.75 | 0.13 | a |  | 3.28 | 0.41 |  |  | nd | - | - |  | 4.53 | 0.30 | a |  | 2.0 | 0.64 |  |
|  |  |  |  |  |  | 0.005 |  |  |  | 0.095 |  |  |  | - |  |  |  | 0.042 |  |  |  | 0.156 |
|  | **IX** |  |  |  |  |  |  |  |  |  |  |  |  |  |  |  |  |  |  |  |  |  |
|  | Leaves Without Cu | qPCR |  | 4.43 | 0.08 | b |  | 4.85 | 0.23 | b |  | 4.94 | 0.11 | b |  | nd | - | - |  | nd | - | - |
|  |  | v-qPCR |  | 4.91 | 0.01 | a |  | 5.33 | 0.10 | a |  | 5.31 | 0.12 | a |  | nd | - | - |  | nd | - | - |
|  |  | PC |  | nd | - | - |  | nd | - | - |  | nd | - | - |  | nd | - | - |  | nd | - | - |
|  |  |  |  |  |  | <0.001 |  |  |  | 0.014 |  |  |  | 0.010 |  |  |  | - |  |  |  | - |
|  | With Cu |  |  |  |  |  |  |  |  |  |  |  |  |  |  |  |  |  |  |  |  |  |
|  |  | qPCR |  | 4.78 | 0.17 | b |  | 4.99 | 0.04 | b |  | 5.01 | 0.22 | b |  | nd | - | - |  | nd | - | - |
|  |  | v-qPCR |  | 5.11 | 0.18 | a |  | 5.36 | 0.11 | a |  | 5.45 | 0.22 | a |  | nd | - | - |  | nd | - | - |
|  |  | PC |  | nd | - | - |  | nd | - | - |  | nd | - | - |  | nd | - | - |  | nd | - | - |
|  |  |  |  |  |  | 0.039 |  |  |  | 0.003 |  |  |  | 0.036 |  |  |  | - |  |  |  | - |
|  | **X** |  |  |  |  |  |  |  |  |  |  |  |  |  |  |  |  |  |  |  |  |  |
|  | Leaves Without Cu | qPCR |  | 5.85 | 0.39 |  |  | 5.17 | 0.12 | a |  | 4.24 | 0.42 |  |  | 5.13 | 0.06 | c |  | 4.17 | 0.41 |  |
|  |  | v-qPCR |  | 5.53 | 0.24 |  |  | 4.84 | 0.32 | a |  | 4.80 | 0.23 |  |  | 5.31 | 0.04 | b |  | 4.67 | 0.33 |  |
|  |  | PC |  | 5.98 | 0.18 |  |  | <LDL |  | b |  | 4.28 | 0.04 |  |  | 5.69 | 0.06 | a |  | 4.67 | 0.17 |  |
|  |  |  |  |  |  | 0.324 |  |  |  | <0.001 |  |  |  | 0.085 |  |  |  | <0.001 |  |  |  | 0.159 |
|  | With Cu |  |  |  |  |  |  |  |  |  |  |  |  |  |  |  |  |  |  |  |  |  |
|  |  | qPCR |  | 5.12 | 0.31 | a |  | 5.42 | 0.14 | a |  | 4.47 | 0.14 | b |  | 5.28 | 0.08 |  |  | 4.22 | 0.21 | b |
|  |  | v-qPCR |  | 4.97 | 0.01 | a |  | 4.55 | 0.62 | a |  | 4.94 | 0.23 | ab |  | 5.34 | 0.17 |  |  | 4.80 | 0.10 | a |
|  |  | PC |  | 5.76 | 0.12 | b |  | <LDL |  | b |  | 5.11 | 0.32 | a |  | 5.55 | 0.25 |  |  | 4.65 | 0.32 | ab |
|  |  |  |  |  |  | 0.015 |  |  |  | <0.001 |  |  |  | 0.040 |  |  |  | 0.232 |  |  |  | 0.050 |
|  |  |  |  |  |  |  |  |  |  |  |  |  |  |  |  |  |  |  |  |  |  |  |
|  | ^a^ Ten field trials (from I to X) were performed. Information of the crop, location, treatment applications, sampling time points is described in Table S1.  ^b^ Mean of three (trials II, III, IV, V, VI, VIII, IX and X) or four (trials I and VII) biological replicates.  ^c^ SD: standard deviation.  ^d^ *p* values < 0.05 indicate significant differences between quantification methods, according to the Tukey’s or t-Student tests. Different letters in the same sampling time points and trial indicate significant differences between quantification methods.  nd: not done.  -: not applicable.  LDL: The lowest limit of detection of plate counting (PC) was 2.7 Log_10_ CFU g^-1^ and it was used for Tukey’s test.  *Amplification signal with Ct value out of the quantification range. Quantification < 3.0 Log_10_ CFU g^-1^ | | | | | | | | | | | | | | | | | | | | | |

**Supplementary Table S6**. Statistical analysis of population dynamics (Log_10_ CFU g^-1^) of *L. plantarum* PM411 estimated by qPCR, v-qPCR and dilution plate counting (PC) at different sampling time points.

|  |  |  |  | **Sampling time point** | | | | | | | | | | | | | | | | | | |
| --- | --- | --- | --- | --- | --- | --- | --- | --- | --- | --- | --- | --- | --- | --- | --- | --- | --- | --- | --- | --- | --- | --- |
|  |  |  |  | **S1** | | |  | **S2** | | |  | **S3** | | |  | **S4** | | |  | **S5** | | |
|  | **Trial ^a^** | **Quantification method** |  | Mean^b^ | SD^c^ | *p* value^d^ |  | mean | SD | *p* value |  | mean | SD | *p* value |  | mean | SD | *p* value |  | mean | SD | *p* value |
| **Apricot** | **I** |  |  |  |  |  |  |  |  |  |  |  |  |  |  |  |  |  |  |  |  |  |
|  |  | qPCR |  | 4.75 | 0.62 | a |  | 5.41 | 1.90 | a |  | 4.43 | 0.33 | a |  | nd | - | -  - |  | nd | - | - |
|  |  | v-qPCR |  | 4.54 | 0.23 | a |  | 3.51 | 0.72 | ab |  | 2.65 | 0.23 | b |  | nd | - |  |  | nd | - | - |
|  |  | PC |  | <LDL | - | b |  | 2.45 | 0.42 | b |  | <LDL | - | b |  | nd | - | - |  | nd | - | - |
|  |  |  |  |  |  | <0.001 |  |  |  | 0.044 |  |  |  | <0.001 |  |  |  | - |  |  |  | - |
|  | **II** |  |  |  |  |  |  |  |  |  |  |  |  |  |  |  |  |  |  |  |  |  |
|  |  | qPCR |  | 4.93 | 0.26 | a |  | 6.21 | 0.26 | a |  | NA | - | - |  | 5.71 | 0.26 | a |  | nd | - | - |
|  |  | v-qPCR |  | NA | - | b |  | 4.55 | 1.24 | ab |  | NA | - | - |  | 5.06 | 0.18 | b |  | nd | - | - |
|  |  | PC |  | <LDL | - | b |  | 2.95 | 0.37 | b |  | <LDL | - | - |  | <LDL | - | c |  | nd | - | - |
|  |  |  |  |  |  | <0.001 |  |  |  | 0.006 |  |  |  | - |  |  |  | <0.001 |  |  |  | - |
| **Grapevine** | **V** |  |  |  |  |  |  |  |  |  |  |  |  |  |  |  |  |  |  |  |  |  |
|  | Berries | qPCR |  | 2.34* | 0.36 | - |  | 3.71 | 0.08 | a |  | 3.71 | 0.37 | a |  | 2.28* | 0.30 | - |  | nd | - | - |
|  |  | v-qPCR |  | NA | - | - |  | NA | - | b |  | NA | - | b |  | NA | - | - |  | nd | - | - |
|  |  | PC |  | <LDL | - | - |  | <LDL | - | b |  | <LDL | - | b |  | <LDL | - | - |  | nd | - | - |
|  |  |  |  |  |  | - |  |  |  | <0.001 |  |  |  | 0.01 |  |  |  | - |  |  |  | - |
|  | **VI** |  |  |  |  |  |  |  |  |  |  |  |  |  |  |  |  |  |  |  |  |  |
|  | Berries | qPCR |  | NA | - | - |  | 4.07 | 0.36 | a |  | 2.66* | 0.67 | a |  | NA | - | - |  | nd | - | - |
|  |  | v-qPCR |  | NA | - | - |  | NA | - | b |  | NA | - | a |  | NA | - | - |  | nd | - | - |
|  |  | PC |  | <LDL | - | - |  | <LDL | - | b |  | <LDL | - | a |  | <LDL | - | - |  | nd | - | - |
|  |  |  |  |  |  | - |  |  |  | <0.001 |  |  |  | 0.668 |  |  |  | - |  |  |  | - |
|  | **VII** |  |  |  |  |  |  |  |  |  |  |  |  |  |  |  |  |  |  |  |  |  |
|  | Leaves | qPCR |  | 6.48 | 0.28 | a |  | NA | - | - |  | nd | - | - |  | nd | - | - |  | nd | - | - |
|  |  | v-qPCR |  | 5.62 | 0.33 | b |  | NA | - | - |  | nd | - | - |  | nd | - | - |  | nd | - | - |
|  |  | PC |  | 3.96 | 0.25 | c |  | <LDL | - | - |  | nd | - | - |  | nd | - | - |  | nd | - | - |
|  |  |  |  |  |  | <0.001 |  |  |  | - |  |  |  | - |  |  |  | - |  |  |  | - |
|  |  |  |  |  |  |  |  |  |  |  |  |  |  |  |  |  |  |  |  |  |  |  |
|  | Berries | qPCR |  | 5.91 | 0.30 | a |  | 4.26 | 1.65 | a |  | 2.89 | 0.25 | ab |  | nd | - | - |  | nd | - | - |
|  |  | v-qPCR |  | 4.58 | 0.42 | b |  | NA | - | ab |  | NA | 0.33 | a |  | nd | - | - |  | nd | - | - |
|  |  | PC |  | 3.76 | 0.40 | c |  | <LDL | - | b |  | <LDL | 0.42 | b |  | nd | - | - |  | nd | - | - |
|  |  |  |  |  |  | <0.001 |  |  |  | 0.060 |  |  |  | 0.008 |  |  |  | - |  |  |  | - |
|  | **VIII** |  |  |  |  |  |  |  |  |  |  |  |  |  |  |  |  |  |  |  |  |  |
|  | Leaves | qPCR |  | 6.33 | 0.25 | a |  | NA | - | - |  | 5.20 | 0.00 | a |  | 5.50 | 0.35 | a |  | 2.37* | 0.21 | a |
|  |  | v-qPCR |  | 5.59 | 0.05 | b |  | NA | - | - |  | 4.47 | 1.12 | a |  | 2.52* | 0.13 | b |  | 2.07* | 0.10 | a |
|  |  | PC |  | 4.04 | 0.20 | c |  | <LDL | - | - |  | 4.75 | 0.94 | a |  | <LDL | - | b |  | <LDL | - | a |
|  |  |  |  |  |  | <0.001 |  |  |  | - |  |  |  | 0.785 |  |  |  | <0.001 |  |  |  | 0.135 |
|  |  |  |  |  |  |  |  |  |  |  |  |  |  |  |  |  |  |  |  |  |  |  |
|  | Berries | qPCR |  | 6.01 | 0.22 | a |  | NA | - | - |  | nd | - | - |  | 5.17 | 0.18 | a |  | 2.46 | 0.71 | a |
|  |  | v-qPCR |  | 3.52 | 0.93 | b |  | NA | - | - |  | nd | - | - |  | 2.28 | 0.10 | b |  | 3.16 | 0.10 | a |
|  |  | PC |  | 3.90 | 0.20 | b |  | <LDL | - | - |  | nd | - | - |  | <LDL | - | b |  | <LDL | - | a |
|  |  |  |  |  |  | 0.003 |  |  |  | - |  |  |  | - |  |  |  | <0.001 |  |  |  | 0.191 |
|  | continue | | | | | | | | | | | | | | | | | | | | | |

**Supplementary Table S6**. (continued)

|  | ^a^ Six field trials (I, II, V, VI, VII, VIII) were performed. Information of the crop, location, treatment applications, sampling time points is described in Table S1.  ^b^ Mean of three (trials II, V, VI, VIII) or four (trials I and VII) biological replicates.  ^c^ SD: standard deviation.  ^d^ *p* values < 0.05 indicate significant differences between quantification methods, according to the Tukey’s or t-Student tests. Different letters in the same sampling time points and trial indicate significant differences between quantification methods.  nd: not done.  -: not applicable.  NA: no amplification. The lowest limit of detection 3 Log_10_ CFU g^-1^ was used for Tukey’s test.  LDL: The lowest limit of detection of plate counting (PC) was 2.7 Log_10_ CFU g^-1^ and it was used for Tukey’s test.  *Amplification signal with Ct value out of the quantification range. Quantification < 3.0 Log_10_ CFU g^-1^ |
| --- | --- |

**Supplementary Table S7**. Tracking of viable cells of *B. velezensis* A17, and *L. plantarum* PM411 in apricot flowers (S1, S2) and immature fruits (S3, S4).

|  |  | **Sampling time point** | | | | | | | | | | | | | | |
| --- | --- | --- | --- | --- | --- | --- | --- | --- | --- | --- | --- | --- | --- | --- | --- | --- |
|  |  | **S1** | | |  | **S2** | | |  | **S3** | | |  | **S4** | | |
|  |  | Mean ^a^ | SD ^b^ | *p* value ^c^ |  | Mean | SD | *p* value |  | Mean | SD | *p* value |  | Mean | SD | *p* value |
| **Apricot** | |  |  |  |  |  |  |  |  |  |  |  |  |  |  |  |
|  |  |  |  |  |  |  |  |  |  |  |  |  |  |  |  |  |
|  | **Trial I ^d^** |  |  |  |  |  |  |  |  |  |  |  |  |  |  |  |
|  | *B. velezensis* A17 | 4.83 | 0.05 | 0.027 |  | 5.57 | 0.26 | 0.003 |  | 3.90 | 0.21 | 0.001 |  | nd | - | - |
|  | *L. plantarum* PM411 | 4.58 | 0.23 |  |  | 3.75 | 0.72 |  |  | 2.68 | 0.23 |  |  | nd | - |  |
|  |  |  |  |  |  |  |  |  |  |  |  |  |  |  |  |  |
|  | **Trial II** |  |  |  |  |  |  |  |  |  |  |  |  |  |  |  |
|  | *B. velezensis* A17 | 4.14 | 0.13 | <0.001 |  | 4.54 | 0.25 | 0.495 |  | 4.78 | 0.24 | <0.001 |  | 5.38 | 0.21 | 0.058 |
|  | *L. plantarum* PM411 | NA | - |  |  | 4.55 | 1.24 |  |  | NA | - |  |  | 5.06 | 0.18 |  |
| ^a^ Viable population levels (Log_10_ CFU g^-1^) estimated by v-qPCR. Mean of three (trial II) or four (trial I) biological replicates  ^b^ SD: standard deviation.  ^c^ *p* values < 0.05 indicate significant differences between A17 and PM411, according to the t-Student test.  ^d^ Two field trials were performed in different growing seasons: trial I 2019 and trial II 2021.  nd: not done.  NA: no amplification. The lowest limit of detection 3 Log_10_ CFU g^-1^ was used for t-Student test. | | | | | | | | | | | | | | | | |

**Supplementary Table S8**. Tracking of viable cells of *B. velezensis* A17, and *L. plantarum* PM411 in grapevine berries.

|  |  | **Sampling time point** | | | | | | | | | | | | | | | | | | |
| --- | --- | --- | --- | --- | --- | --- | --- | --- | --- | --- | --- | --- | --- | --- | --- | --- | --- | --- | --- | --- |
|  |  | **S1** | | |  | **S2** | | |  | **S3** | | |  | **S4** | | |  | **S5** | | |
|  |  | Mean ^a^ | SD ^b^ | *p* value ^c^ |  | Mean | SD | *p* value |  | Mean | SD | *p* value |  | Mean | SD | *p* value |  | Mean | SD | *p* value |
| **Grapevine - berries** | |  |  |  |  |  |  |  |  |  |  |  |  |  |  |  |  |  |  |  |
|  |  |  |  |  |  |  |  |  |  |  |  |  |  |  |  |  |  |  |  |  |
|  | **Trial V ^d^** |  |  |  |  |  |  |  |  |  |  |  |  |  |  |  |  |  |  |  |
|  | *B. velezensis* A17 | 3.10 | 0.43 | <0.351 |  | 3.74 | 0.32 | 0.008 |  | 3.38 | 0.19 | 0.013 |  | 3.57 | 0.41 | 0.035 |  | nd | - | - |
|  | *L. plantarum* PM411 | NA | - |  |  | NA | - |  |  | NA | - |  |  | NA | - |  |  | nd | - |  |
|  |  |  |  |  |  |  |  |  |  |  |  |  |  |  |  |  |  |  |  |  |
|  | **Trial VI** |  |  |  |  |  |  |  |  |  |  |  |  |  |  |  |  |  |  |  |
|  | *B. velezensis* A17 | 3.78 | 0.38 | 0.012 |  | 3.93 | 0.28 | <0.001 |  | 3.36 | 0.41 | <0.001 |  | 3.20 | 0.17 | <0.001 |  | nd | - | - |
|  | *L. plantarum* PM411 | NA | - |  |  | NA | - |  |  | NA | - |  |  | NA | - |  |  | nd | - |  |
|  |  |  |  |  |  |  |  |  |  |  |  |  |  |  |  |  |  |  |  |  |
|  | **Trial VII** |  |  |  |  |  |  |  |  |  |  |  |  |  |  |  |  |  |  |  |
|  | *B. velezensis* A17 | 4.68 | 0.34 | 0.359 |  | 4.16 | 0.12 | <0.001 |  | 3.84 | 0.14 | <0.001 |  | nd | - | - |  | nd | - | - |
|  | *L. plantarum* PM411 | 4.58 | 0.42 |  |  | NA | - |  |  | NA | - |  |  | nd | - |  |  | nd | - |  |
|  |  |  |  |  |  |  |  |  |  |  |  |  |  |  |  |  |  |  |  |  |
|  | **Trial VIII** |  |  |  |  |  |  |  |  |  |  |  |  |  |  |  |  |  |  |  |
|  | *B. velezensis* A17 | 4.26 | 0.06 | 0.123 |  | 3.99 | 0.01 | <0.001 |  | nd | - | - |  | 4.14 | 0.16 | 0.011 |  | 3.35 | 0.11 | 0.043 |
|  | *L. plantarum* PM411 | 3.52 | 0.93 |  |  | NA | - |  |  | nd | - |  |  | 2.28 | 0.88 |  |  | 3.16 | 0.10 |  |
|  |  |  |  |  |  |  |  |  |  |  |  |  |  |  |  |  |  |  |  |  |
| ^a^ Viable population levels (Log_10_ CFU g^-1^) estimated by v-qPCR. Mean of three (trial V, VI, VIII) or four (trial VII) biological replicates  ^b^ SD: standard deviation.  ^c^ *p* values < 0.05 indicate significant differences between A17 and PM411, according to the t-Student test.  ^d^ Four field trials were performed in different growing seasons: trial V and VII 2019 and trial VI and VIII 2020.  nd: not done.  NA: no amplification. The lowest limit of detection 3 Log_10_ CFU g^-1^ was used for t-Student test. | | | | | | | | | | | | | | | | | | | | |

**Supplementary Table S9**. Tracking of viable cells of *B. velezensis* A17 and *L. plantarum* PM411 in grapevine leaves.

|  |  | **Sampling time point** | | | | | | | | | | | | | | | | | | |
| --- | --- | --- | --- | --- | --- | --- | --- | --- | --- | --- | --- | --- | --- | --- | --- | --- | --- | --- | --- | --- |
|  |  | **S1** | | |  | **S2** | | |  | **S3** | | |  | **S4** | | |  | **S5** | | |
|  |  | Mean ^a^ | SD ^b^ | *p* value ^c^ |  | Mean | SD | *p* value |  | Mean | SD | *p* value |  | Mean | SD | *p* value |  | Mean | SD | *p* value |
| **Grapevine - leaves** | |  |  |  |  |  |  |  |  |  |  |  |  |  |  |  |  |  |  |  |
|  |  |  |  |  |  |  |  |  |  |  |  |  |  |  |  |  |  |  |  |  |
|  | **Trial VII ^d^** |  |  |  |  |  |  |  |  |  |  |  |  |  |  |  |  |  |  |  |
|  | *B. velezensis* A17 | 5.48 | 0.16 | 0.238 |  | 5.43 | 0.30 | <0.001 |  | nd | - | - |  | nd | - | - |  | nd | - | - |
|  | *L. plantarum* PM411 | 5.62 | 0.33 |  |  | NA | - |  |  | nd | - |  |  | nd | - |  |  | nd | - |  |
|  |  |  |  |  |  |  |  |  |  |  |  |  |  |  |  |  |  |  |  |  |
|  | **Trial VIII** |  |  |  |  |  |  |  |  |  |  |  |  |  |  |  |  |  |  |  |
|  | *B. velezensis* A17 | nd | - | - |  | 4.26 | 0.07 | <0.001 |  | 5.05 | 0.18 | 0.214 |  | 4.69 | 0.14 | <0.001 |  | 4.40 | 0.20 | 0.006 |
|  | *L. plantarum* PM411 | 5.59 | 0.05 |  |  | NA | - |  |  | 4.47 | 1.12 |  |  | 2.52 | 0.13 |  |  | 2.07 | 0.10 |  |
|  |  |  |  |  |  |  |  |  |  |  |  |  |  |  |  |  |  |  |  |  |
|  | **Trial IX** |  |  |  |  |  |  |  |  |  |  |  |  |  |  |  |  |  |  |  |
|  | *B. velezensis* A17 | 4.91 | 0.01 | 0.057 |  | 5.33 | 0.10 | 0.372 |  | 5.31 | 0.13 | 0.198 |  | nd | - | - |  | nd | - | - |
|  | *B. velezensis* A17 + Cu | 5.11 | 0.18 |  |  | 5.36 | 0.11 |  |  | 5.45 | 0.22 |  |  | nd | - |  |  | nd | - |  |
|  |  |  |  |  |  |  |  |  |  |  |  |  |  |  |  |  |  |  |  |  |
|  | **Trial X** |  |  |  |  |  |  |  |  |  |  |  |  |  |  |  |  |  |  |  |
|  | *B. velezensis* A17 | 5.53 | 0.24 | 0.041 |  | 4.84 | 0.32 | 0.256 |  | 4.80 | 0.24 | 0.252 |  | 5.31 | 0.04 | 0.396 |  | 4.67 | 0.33 | 0.276 |
|  | *B. velezensis* A17 + Cu | 4.97 | 0.01 |  |  | 4.55 | 0.62 |  |  | 4.94 | 0.23 |  |  | 5.34 | 0.17 |  |  | 4.80 | 0.10 |  |
|  |  |  |  |  |  |  |  |  |  |  |  |  |  |  |  |  |  |  |  |  |
| ^a^ Viable population levels (Log_10_ CFU g^-1^) estimated by v-qPCR. Mean of three (trial VIII, IX, X) or four (trial VII) biological replicates  ^b^ SD: standard deviation.  ^c^ *p* values < 0.05 indicate significant differences between A17 and PM411 or A17 with and without Cu, according to the t-Student test.  ^d^ Four field trials were performed in different growing seasons: trial VII and IX 2019 and trial VIII and X 2020.  nd: not done.  NA: no amplification. The lowest limit of detection 3 Log_10_ CFU g^-1^ was used for t-Student test. | | | | | | | | | | | | | | | | | | | | |
